# Highly Pathogenic Avian Influenza A(H5N1) Virus Struck Migratory Birds in China in 2015

**DOI:** 10.1038/srep12986

**Published:** 2015-08-11

**Authors:** Yuhai Bi, Zhenjie Zhang, Wenjun Liu, Yanbo Yin, Jianmin Hong, Xiangdong Li, Haiming Wang, Gary Wong, Jianjun Chen, Yunfeng Li, Wendong Ru, Ruyi Gao, Di Liu, Yingxia Liu, Boping Zhou, George F. Gao, Weifeng Shi, Fumin Lei

**Affiliations:** 1 CAS Key Laboratory of Pathogenic Microbiology and Immunology, Institute of Microbiology, Chinese Academy of Sciences, Beijing 100101, China; 2 Shenzhen Key Laboratory of Pathogen and Immunity, Shenzhen Third People’s Hospital, Shenzhen 518112, China; 3 Institute of Pathogen Biology, Taishan Medical College, Taian 271016, China; 4 CAS Key Laboratory of Zoological Systematics and Evolution, Institute of Zoology, Chinese Academy of Sciences, Beijing 100101, China; 5 College of Animal Science and Veterinary Medicine, Qingdao Agricultural University, Qingdao 266109, China; 6 Center for Influenza Research and Early-warning (CASCIRE), Chinese Academy of Sciences, Beijing 100101, China; 7 Beijing Key Laboratory of Plant Genetic Resources and Low-carbon Environmental Biotechnology, Capital Normal University, Beijing 100048, China; 8 State Key Laboratory for Agrobiotechnology, China Agricultural University, Beijing 100193, China; 9 College of Animal Science and Veterinary Medicine, Shandong Agricultural University, Taian 271018, China; 10 CAS Key Laboratory of Special Pathogens and Biosafety, Wuhan Institute of Virology, Chinese Academy of Sciences, Wuhan 430071, China; 11 National Urban Wetland Park of Sanmenxia Swan Lake, Sanmenxia 472000, China; 12 Collaborative Innovation Center for Diagnosis and Treatment of Infectious disease, Zhejiang University, Hanzhou 310003, China

## Abstract

Approximately 100 migratory birds, including whooper swans and pochards, were found dead in the Sanmenxia Reservoir Area of China during January 2015. The causative agent behind this outbreak was identified as H5N1 highly pathogenic avian influenza virus (HPAIV). Genetic and phylogenetic analyses revealed that this Sanmenxia H5N1 virus was a novel reassortant, possessing a Clade 2.3.2.1c HA gene and a H9N2-derived PB2 gene. Sanmenxia Clade 2.3.2.1c-like H5N1 viruses possess the closest genetic identity to A/Alberta/01/2014 (H5N1), which recently caused a fatal respiratory infection in Canada with signs of meningoencephalitis, a highly unusual symptom with influenza infections in humans. Furthermore, this virus was shown to be highly pathogenic to both birds and mammals, and demonstrate tropism for the nervous system. Due to the geographical location of Sanmenxia, these novel H5N1 viruses also have the potential to be imported to other regions through the migration of wild birds, similar to the H5N1 outbreak amongst migratory birds in Qinghai Lake during 2005. Therefore, further investigation and monitoring is required to prevent this novel reassortant virus from becoming a new threat to public health.

The H5N1 subtype of highly pathogenic avian virus (HPAIV), initially identified during 1996 in China, infected 18 humans with 6 deaths during 1997 in Hong Kong^1^. This virus was highly pathogenic in chickens and humans, and posed a significant threat to public health. As of 31 March 2015, H5N1 virus caused at least 826 laboratory-confirmed human infections, including 440 deaths across 16 countries^2^. Direct or indirect contact with diseased poultry is the primary route of HPAIV infections in humans^3^. Despite measures to prevent HPAIV spread by vaccination or the culling of infected birds, several H5 influenza subtypes are already prevalent in Asia, Europe, and Africa^4^. Wild waterfowl are currently regarded as a natural reservoir for avian influenza viruses (AIV)^5^,^6^,^7^,^8^, and contribute to the geographical spread of AIV via long-distance migration, before transmission to domestic poultry^7^,^9^,^10^,^11^.

Apart from sporadic cases, widespread infections and deaths of wild birds from HPAIV infection have never been reported before 2005. However, a HPAIV of the H5N1 subtype killed thousands of bar-headed geese (*Anser indicus*), great black-headed gulls (*Larus ichthyaetus*), and brown-headed gulls (*Larus brunnicephalus*) in Qinghai Lake, China during May 2005^9^,^12^. In 2006, a Qinghai-like Clade 2.2 virus re-emerged in Qinghai Lake and caused more infections in wild birds, including bar-headed geese and great black-headed gulls. The Qinghai-like Clade 2.2 virus was found to possess a high genetic relationship with viruses isolated from other countries on the migratory flyway of wild birds^4^, suggesting that the migration of wild birds played an important role in circulating H5N1 HPAIV viruses between the different avian populations. The virus eventually spread into other parts of Asia, as well as Europe and Africa, resulting in the deaths of millions of birds, and hundreds of cases in humans^9^,^13^,^14^,^15^,^2^.

In 2009, another novel H5N1 virus (Clade 2.3.2) emerged and caused an outbreak resulting in the deaths of more than 200 wild birds, including great cormorants (*Phalacrocorax carbo*), brown-headed gulls, great black-headed gulls, great-crested grebes (*Podiceps cristatus*), bar-headed geese, ruddy shelducks (*Tadorna ferruginea*), and common coots (*family Rallidae*) in Qinghai Lake^16^,^17^. Further analysis showed that the viruses belonged to Clade 2.3.2.1, which had first emerged in whooper swans (*Cygnus cygnus*) during 2008 in Japan^16^,^17^. This virus subsequently re-emerged in Mongolia during 2009–2010 in the bar-headed geese, whooper swans, common goldeneyes (*Bucephala clangula)*, and ruddy shelducks^18^; in Russia during 2009–2010 with the great-crested grebes and great black-headed gulls^18^; as well as in Korea in mallard ducks, and Japan including whooper swans, greater scaups (*Aythya marila)*, pintails (*Anas acuta*), peregrine falcons (*Falco peregrinus)*, tufted ducks (*Aythya fuligul*a), common pochards (*Aythya ferina*), great crested grebes (*Podiceps cristatus*), tundra swans (*Cygnus columbianus*), ural owls (*Strix uralensis*), mandarin ducks (*Aix galericulata*), and goshawks (*Accipiter gentilis*) during 2010–2011^13^,^19^. Interestingly, the novel Clade 2.3.2.1 virus circulated mainly in wild waterfowl and was only occasionally isolated in domestic poultry.

In January of 2015, the deaths of approximately 100 migratory wild birds (mainly whooper swans and pochards) in the Sanmenxia Reservoir Area of China were investigated. Located approximately 1150 kilometers (km) east of Qinghai Lake, this location is an important wintering habitat for whooper swans and various species of wild ducks including pochards (*Aythya ferina*) migrating to China from Mongolia and Siberia. The deaths sparked concerns of another HPAIV outbreak being spread by migratory wild birds.

To investigate this outbreak, samples were taken from sick and dead wild birds, and H5N1 virus was identified as the causative agent. In this study the gene evolution, phylogenetics, virulence to chickens and mammals, as well as receptor binding properties of these H5N1 viruses are presented, in addition to its potential spread along the migratory flyway of wild birds.

## Results

### Virus isolation

Samples were collected from sick or dead birds, in addition to the surrounding environment, in the Sanmenxia Reservoir Area of China on January 4^th^–5^th^, 2015. All samples were inoculated in 10-day-old, embryonated chicken eggs, which were found to have died within 24~41 hours after inoculation. Allantoic fluids tested positive by a hemagglutination (HA) assay with titers between 128–256 reciprocal dilutions. These suggested that this virus is highly pathogenic to poultry. H5N1 influenza virus was confirmed and Newcastle disease virus (NDV) was ruled out by HA inhibition (HI) assay and RT-PCR (specific primer sequences for both viruses are available from the corresponding author upon request). In addition, no bacteria were detected in the organs and tissues of the sick or dead birds when the samples were plated onto LB agar.

A total of 12 influenza A (H5N1) viruses were isolated, with ten isolates from whooper swans, one from a dead pochard, and one isolate from 12 environmental samples containing the feces of apparently healthy birds.

### Genetic identity analysis

The H5 and N1 sequences of the 12 viruses were found to possess high genetic identities with each other. To avoid redundancy, six of 12 viruses (Tables 1 and 2) were selected to further analyze the gene evolution of the Sanmenxia H5N1 viruses isolated from wild birds. Genetic analysis showed that all eight gene segments of the novel H5N1 viruses shared between 99.3~100% nucleotide identity with each other.

Sequence alignments and BLAST search in the GISAID and NCBI Flu database showed that the genomes of the Sanmenxia H5N1 viruses showed the highest genetic homology to a previously described H5N1 virus (A/Alberta/01/2014, Alberta2014). Alberta2014 was responsible for causing the first human H5N1 infection in North America, and was likely an imported case from Asia^20^. The nucleotide identities of the novel virus isolates with Alberta2014 ranged between 98.7 to 99.6%, and protein identities ranged between 98.2~100%. These new isolates also possessed high nucleotide (91.6~98.7%) and amino acid (97.6~100%) identities to another mammalian H5N1 strain (A/tiger/Jiangsu/01/2013, Tiger2013), which was isolated from a tiger that had died from respiratory failure in 2013^21^ (Table 1).

### Phylogenetic analysis

**Figure 1:**
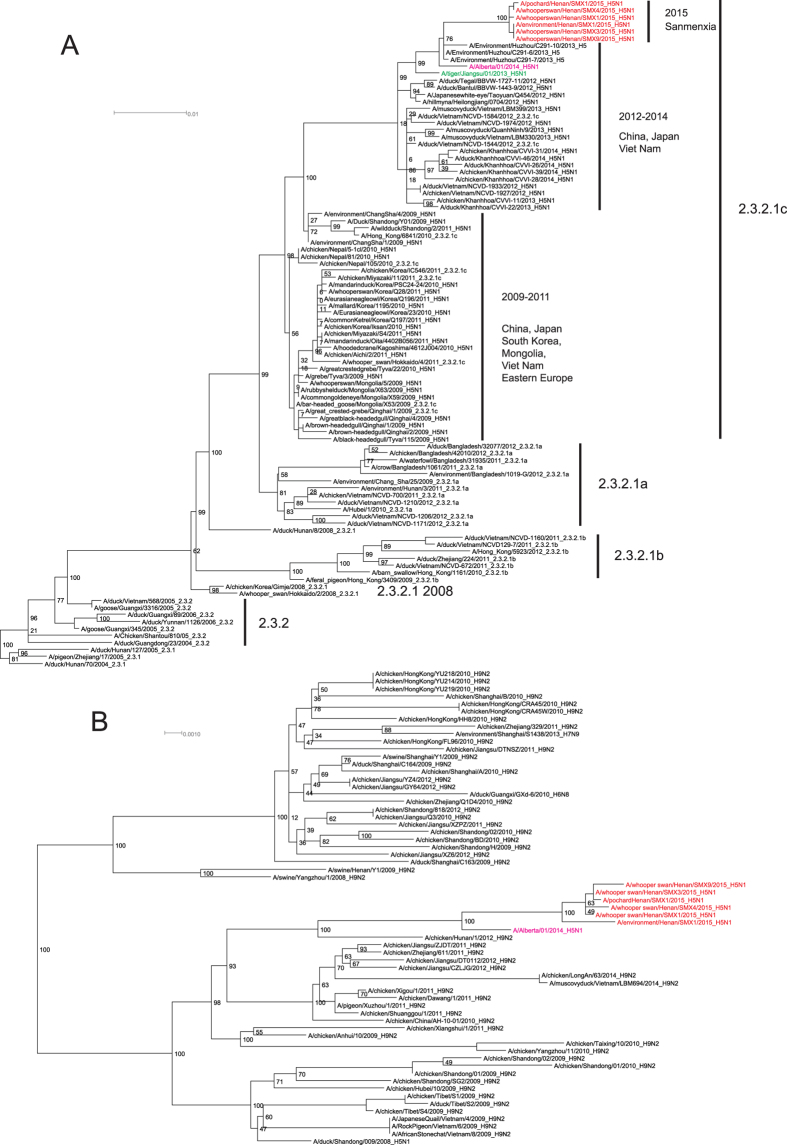
Phylogenetic analyses of the HA and PB2 gene sequences from Sanmenxia Clade 2.3.2.1c H5N1 isolates. The reference sequences were downloaded from the GISAID and GenBank database. All of the phylogenetic analyses were performed using Raxml, with 1000 bootstrap replicates. (**A)** phylogenies of Clade 2.3.2 HA gene for H5N1 HPAIVs. (**B)** phylogenetic tree constructed using the PB2 gene sequences. Segments of the whooper swan-H5N1, A/Alberta/01/2014(H5N1), and A/tiger/Jiangsu/01/2013(H5N1) viruses are colored in red, pink and green, respectively.

To further analyze the genetic evolution of these novel H5N1 viruses, phylogenetic analysis of the whole genomes was performed with reference H5N1 strains. The HA gene of the new isolates was found to belong to Clade 2.3.2.1c (Figs 1A and S1). Notably, the topology of Clade 2.3.2.1c formed a “ladder-like” phylogenetic structure, and H5N1 strains causing the Sanmenxia outbreak formed a novel independent “ladder” (Fig. 1A). The other distinct “ladder-like” branches include the 2009–2011 sub-clade circulating in Asia and Eastern Europe, and the 2012–2014 sub-clade circulating in Eastern Asia. Therefore, Clade 2.3.2.1c has been circulating in Eastern Asia since 2009, undergoing a lineage replacement every 2–3 years. However, the gap between the sub-clade 2.3.2.1 in 2008 and the recent 2015 sub-clade is quite large, as indicated by branch length (Fig. 1A), but it is currently unknown whether these variations in gene sequences influence viral antigenicity/pathogenicity.

Consistent with results obtained from the comparison of sequence identities, isolates from the 2015 outbreak were most closely related to a H5 lineage circulating in China from 2013 in the HA phylogenetic tree, including Alberta2014 and Tiger2013 (Fig. 1A). Similar topologies were also observed in the phylogenetic trees constructed using the NA, NP, MP, NS, PA, and PB1 gene sequences (Figs S2–S7).

The PB2 genes of the novel isolates and Alberta2014 were found to cluster together with previously circulating H9N2 viruses in China (Fig. 1B). Interestingly, the PB2 gene of Tiger2013 was also not derived from the H5 lineage (Fig. S8), but rather it belonged to an AIV gene pool that mainly existed in wild birds (Fig. S8), confirming the results of a previous study^21^. Therefore, the novel isolates from Sanmenxia and Alberta2014 belong to the same genotype, with the HA gene from Clade 2.3.2.1c, and the PB2 gene from H9N2 origin. The Tiger2013 isolate belongs to a different genotype, with the HA gene from Clade 2.3.2.1c, and the PB2 gene from an AIV gene pool.

### Genetic signature analysis

The genetic signatures of the Sanmenxia Clade 2.3.2.1c-like H5N1 isolates were analyzed for their potential biological characteristics in order to lay the groundwork for future experiments regarding virus pathogenicity, host adaptation and drug sensitivity. The results showed that the novel isolates identified in this outbreak possessed multiple basic amino acids at the cleavage sites of HA, RERRRKR/G (Table 2), which is the typical molecular signature for HPAIVs^22^. In addition, the HA proteins of these isolates had the amino acids Q226 and G228 (H3 numbering) in the receptor-binding site. This suggested that these viruses favor binding to avian-like receptors, but the N158D and T160A substitutions in these viruses may enhance their capacity to also bind human-type receptors^23^,^24^,^25^. Amino acids Q591, E627 and D701 were observed in the PB2 protein, suggesting that these novel H5N1 isolates are not yet fully adapted to infect mammals.

In light of the close genetic relationship between the H5N1 viruses isolated from wild birds and mammals, the genetic signatures of these viruses were also compared with Alberta2014 and Tiger2013. Interestingly, there are only five amino acid mutations in the HA proteins between H5N1 virus isolates from wild birds and human/tiger (Tables 2 and 3). The D225R substitution was found only in Alberta2014, which may influence viral receptor binding properties, according to the results of a previous study^26^.

Furthermore, the NA stalk of these novel H5N1 viruses possessed a 20 amino acid deletion (positions 49 to 68). Past studies had shown that AIVs with this deletion demonstrated better adaptation to terrestrial birds after they were introduced from wild birds, and showed increased virulence in mammals^27^,^28^. Moreover, these H5N1 isolates had a C-terminal (aa 58–90) truncated PB1-F2 protein with only 57 amino acids, which has also been shown to contribute to their virulence in mammals^29^,^30^. Hence, the novel H5N1 isolates may be highly pathogenic to mammals based on the molecular characteristics on NA stalk and PB1-F2, even though they possess the avian adaptation residues with Q591, E627 and D701 on the PB2 protein.

The NA-inhibitor resistance mutation H274Y was not found in the NA antigen of these novel H5N1 reassortants. However, the V27I substitution was observed in the M2 protein, indicating that these H5N1 isolates are sensitive to NA inhibitors, but may be resistant to M2 inhibitors^2^.

### Pathogenicity in birds and mice

To further characterize the virulence of these new H5N1 viruses, one representative strain (whooper swan/HN/SMX4/2015) was selected for studies in chickens and mice. All ten specific-pathogen free (SPF) chickens inoculated with whooper swan/HN/SMX4/2015 died 1~3 days post-infection (d.p.i.) and the intravenous pathogenicity index (IVPI) was 2.79 (Table 4), indicating that these novel H5N1 viruses are HPAIV.

**Figure 2:**
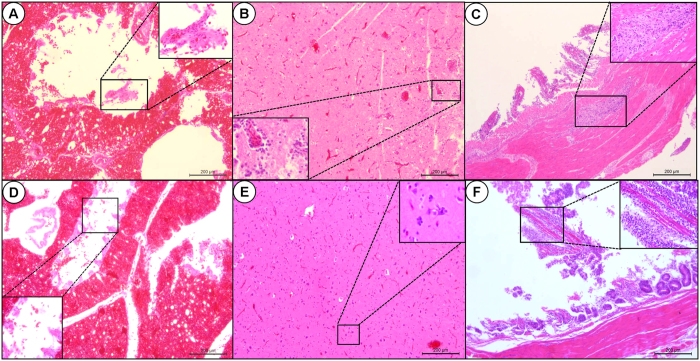
Histopathologic analysis in lung, brain, and small intestine of the dead whooper swan and pochard naturally infected by the H5N1 virus. Representative pictures of the lung (**A**,**D**), brain (**B**,**E**), and small intestine (**C**,**F**) of the whooper swan (**A–C**) and pochard (**D–F**) infected by the Sanmenxia Clade 2.3.2.1c H5N1 isolate. Scale bar = 200 μm. Bronchial epithelial cell desquamation, congestion and hemorrhage are found in the lungs (**A,D**); Inflammatory cells infiltrate around blood vessel and nerve cells are found in the brains (**B,E**). Intestinal villus desquamation, lymphatic tissue cells neorobiosis and disaggregation under mucosa are found in the small intestines (**C,F**).

In addition, the novel H5N1 virus was found to cause severe pathological lesions in the non-surviving wild whooper swans and the pochard. Viral encephalitis, severe lung damage with congestion and hemorrhage, and necroticans enteritis can be readily observed from the histopathology results (Fig. 2).

**Figure 3:**
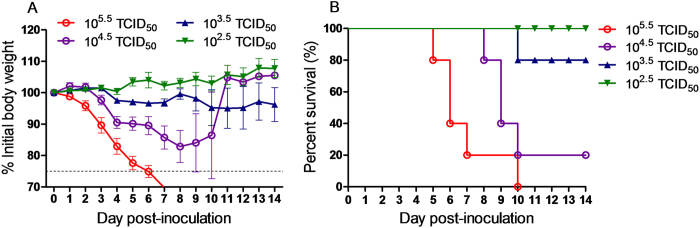
Pathogenicity of the Sanmenxia H5N1 virus in mice. Mice were infected by 10-fold serial dilutions of Ws/HN/SMX4/15(H5N1) virus. (**A)** Average percentage weight change. (**B)** Kaplan-Meier survival curve.

Furthermore, whooper swan/HN/SMX4/2015 was also shown to be highly pathogenic in mice, with a median lethal dose (MLD_50_) of 10^3.0^ TCID_50_ (median tissue culture infective dose) (Fig. 3 and Table 4). The virus not only replicated in the lungs during 3~7 d.p.i. with high lung virus titers (LVTs), but was found to be present in the brains starting at 5 d.p.i. Interestingly, LVTs peaked at 5 d.p.i. before gradually decreasing, whereas the peak of virus replication in the brain was at 7 d.p.i. (Table 4). This suggests that the virus first replicated in the target organs (lungs), before bypassing the blood brain barrier to infect the brain.

### Receptor binding properties

**Figure 4:**
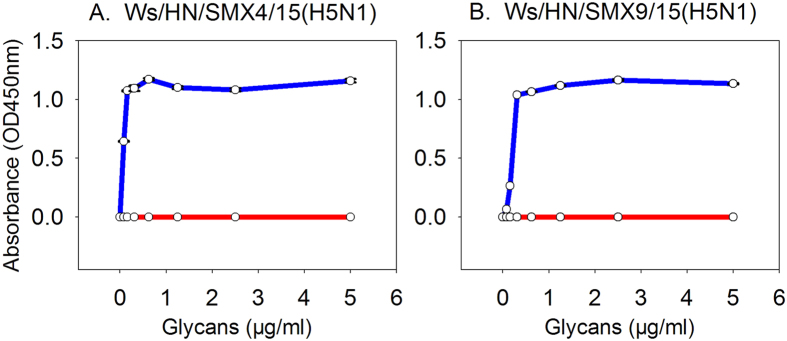
Receptor binding properties of the Sanmenxia H5N1 virus. Solid-phase binding assay for (**A)** Ws/HN/SMX4/15(H5N1) and (**B)** Ws/HN/SMX9/15(H5N1) viruses to both α2,3-linked (3’SLNLN) (colored in blue) and α2,6-linked sialylglycan receptors (6’SLNLN) (colored in red), respectively. The data presented is the mean ± standard deviation (S.D.).

In light of the highly pathogenic nature of the new H5N1 isolates, as well as the high genetic identities between these viruses and H5N1 of human origin, potential risks of these novel H5N1 viruses to humans was evaluated by a solid-phase receptor binding assay. The new H5N1 isolates were found to bind preferentially to the avian α-2,3-linked (3′SLNLN) receptor over the human α-2,6-linked (6′SLNLN) sialylglycan receptor (Fig. 4), suggesting that these novel isolates currently have a low risk for human infections.

## Discussion

Genetic and phylogenetic analyses showed that this outbreak in whooper swans, pochards, and other wild ducks in the Sanmenxia Reservoir Area was caused by a H5N1 virus. These viruses were shown to be novel reassortants, possessing a Clade 2.3.2.1c HA gene and an H9N2-derived PB2 gene, and had the highest genetic homology with Alberta2014 and Tiger2013. The HA protein of these novel isolates also have the N158D and T160A amino acid substitutions, which were associated with at least two confirmed mammalian infections in the past^23^,^24^ and highlights the potential risk to public health.

The novel isolates were found to be highly pathogenic to chickens and mice, and virus isolated from mouse brains after challenge. It should be noted that meningoencephalitis was also observed in the fatal human case with Alberta2014, an unusual outcome for infections with H5N1 HPAIV in humans^20^. Neurological symptoms were also noted in the non-surviving tiger infected with Tiger2013, with the heart, liver, spleen, lung, kidney, aquae pericardii, and cerebrospinal fluid all positive for H5N1 virus as detected by real-time RT-PCR^21^. This suggests that the novel Sanmenxia Clade 2.3.2.1c-like H5N1 viruses possesses tropism for the nervous system in several mammal species, and could pose a significant threat to humans if these viruses develop the ability to bind human-type receptors more effectively.

**Figure 5:**
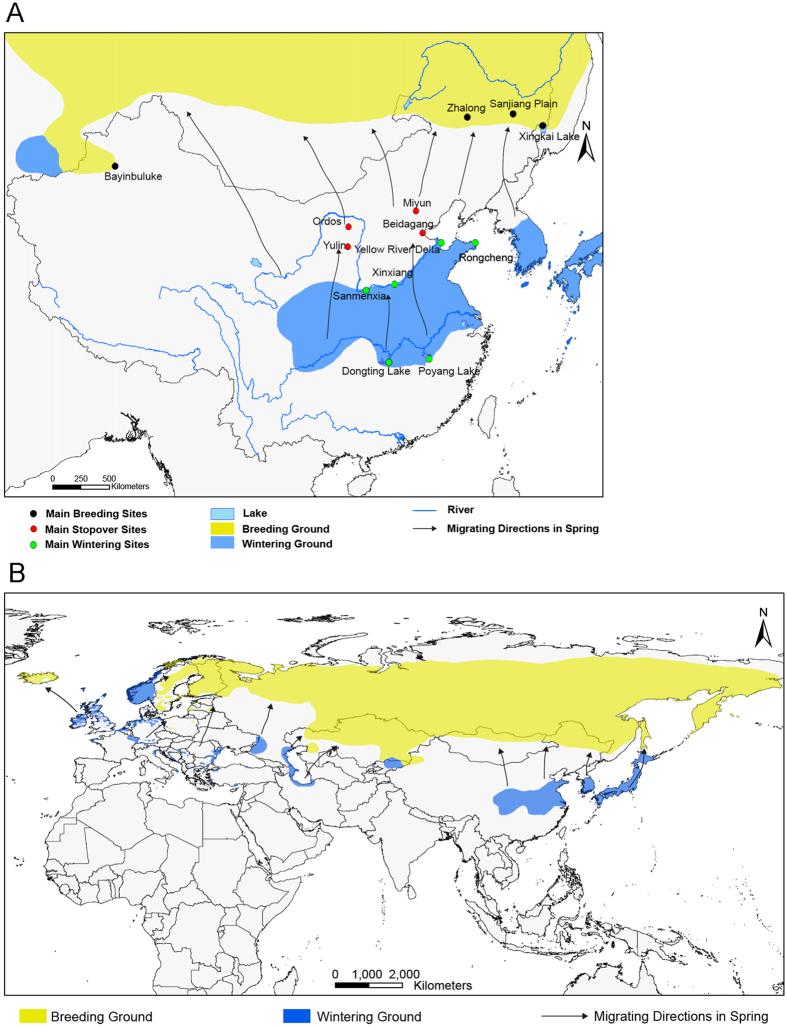
The migration routes of whooper swan (*Cygnus cygnus*). The migratory routes of whooper swan in China (**A**) and worldwide (**B**) were mapped by the ArcGIS Desktop 10.2 software.

**Figure 6:**
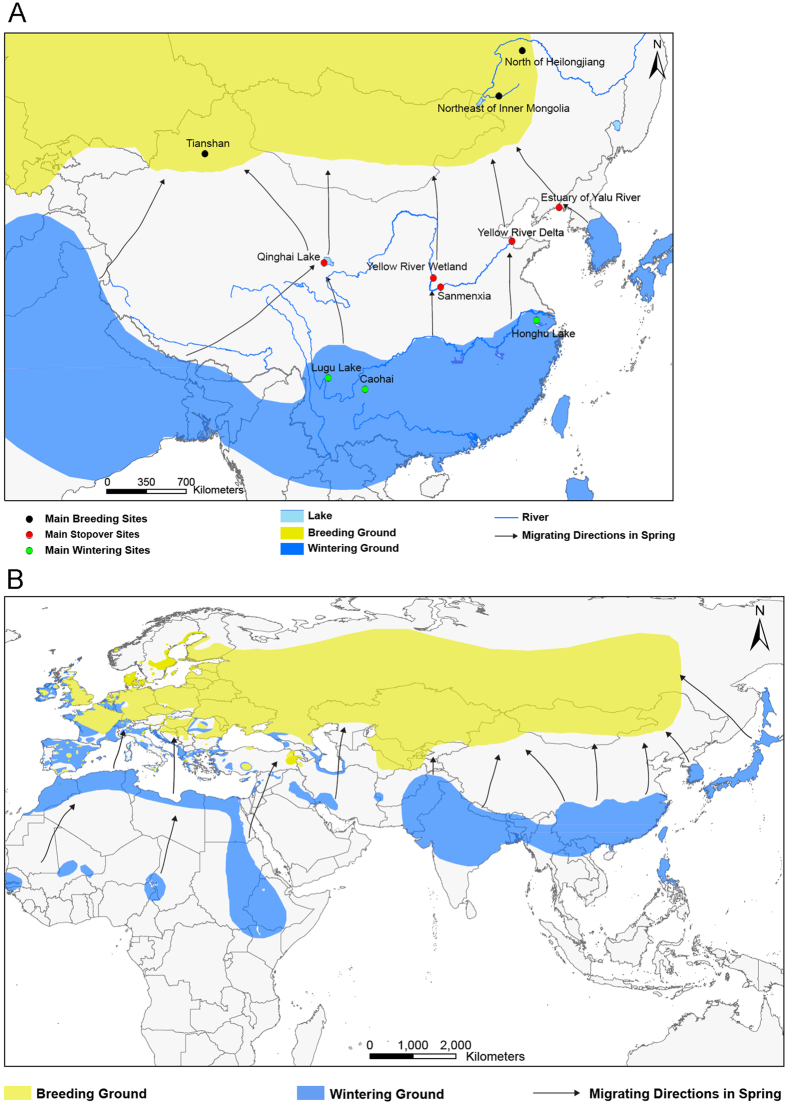
The migration routes of pochard (*Aythya ferina*). The migratory routes of pochard in China (**A**) and worldwide (**B**) were mapped by the ArcGIS Desktop 10.2 software.

No domestic poultry breeding farms exist within 3 km of the Sanmenxia Reservoir Area and the majority of the Clade 2.3.2.1c strains are isolated from migratory birds (Fig. 1A). Therefore, the Sanmenxia Clade 2.3.2.1c-like H5N1 virus is likely spread by the long-distance migration of wild birds, especially ducks, which might have been infected by the virus from domestic poultry during stopover sites in their migration^6^,^10^,^11^,^31^. Whooper swans are known to breed in northern Eurasia, and winter in Europe and eastern Asia, such as China, Korea, and Japan^32^. There are approximately 20 000 whooper swans wintering in China, and more than half of these animals winter in the Sanmenxia Reservoir Area along with various ducks from the East Asian/Australasian and Central Asian flyways^33^. These birds arrive in the Sanmenxia Reservoir Area during early October for wintering, and then fly back to Mongolia and Siberia for breeding next spring (Figs 5 and 6).

It is believed that overlapping migratory flyways help circulate H5 HPAIVs amongst different bird species, and allow the spread of the virus across continents^8^. Recently sequential outbreaks of the H5N8 virus in domestic poultry in China, Korea, and Japan during 2010–2015^34^,^35^, Europe in 2014–2015^36^, and North America in 2014–2015^4^,^37^,^38^ were considered to have occurred due to waterfowl migration^36^,^37^,^39^. Therefore, the migration of wild birds plays an important role in the transmission and spread of H5 HPAIVs, posing a severe risk to animal and human health. Further investigation is required to monitor the evolution and transmission of these novel Sanmenxia Clade 2.3.2.1c-like H5N1 isolates, in order to prevent their spread to other countries and avoiding a repeat of what had happened in the past with circulating Qinghai-like Clades 2.2 and 2.3.2 H5N1, and Clade 2.3.4.4 H5N8 viruses^7^,^9^,^15^,^16^,^19^,^36^,^37^,^40^,^41^.

## Materials and Methods

### Virus isolation and gene sequencing

The oropharyngeal and cloacal swabs, tissues and organs of the sick or dead birds, and feces from apparently healthy birds were collected for virus isolation. Swab samples and tissues were maintained at 4 ^°^C in viral-transport medium and sample tubes, respectively. Before inoculation, swab samples with viral-transport medium were thoroughly mixed. The tissues and organs were homogenized using a Qiagen TissueLyser II machine (30 cycles/s; 4 min) in 1 ml of cold PBS under sterile conditions. The solid debris in the swab and tissue samples were pelleted by centrifugation at 5,000 × g for 10 min. The supernatants were then inoculated in 10-day-old chicken embryos for 72 hours at 37 °C.

The hemagglutination (HA) and hemagglutination inhibition (HI) assay with H5N1 AIV and Newcastle disease virus (NDV) antigens and their antisera was performed according to WHO standard protocols^42^. Viral RNA from the HA titer-positive allantoic fluid was extracted using TRIZOL (Invitrogen) according to manufacturer instructions. All gene segments were amplified using *Ex Taq*™ DNA polymerase (Takara) with the improved primers^43^, and sequenced by the ABI 3730XL automatic DNA analyzer.

### Genetic and phylogenetic analysis

The gene and amino acid sequences were edited using DNAMAN (Version 7.0) and BioEdit (Version 7.0.5.2). Referenced sequences were downloaded from the NCBI (http://www.ncbi.nlm.nih.gov) and GISAID (www.gisaid.org) databases via the online Blast search. Multiple sequence alignment was performed using Muscle^44^. Maximum-likelihood phylogenetic trees were inferred with the software RAxML^45^ under the GAMMAGTR model with 1000 bootstraps.

### Histopathologic analysis

The brain, lung, small intestine, spleen, kidney, and liver of non-surviving wild birds were fixed in 10% phosphate-buffered formalin, embedded in paraffin, cut into 5-mm-thick serial sections, and stained with hematoxylin and eosin (H&E) for histopathological evaluation as previously described^46^.

### Studies in chickens

To determine the pathogenicity of these novel virus isolates, the IVPI was determined according to the recommendations of the Manual of Diagnostic Tests and Vaccines for Terrestrial Animals 2014^47^. Fresh infective allantoic fluid with a HA titer of 128 was diluted 1/10 in sterile isotonic saline. 0.1 ml of the diluted virus was used for an intravenous challenge in ten 6-week-old SPF White Leghorn chickens.

Birds were examined at 24-hour intervals for 10 days. At each observation, each bird was scored 0 if normal, 1 if sick, 2 if severely sick, and 3 if dead. The judgment of sick and severely sick birds is a subjective clinical assessment. Typically, ‘sick’ birds demonstrate one of the following signs and those that are ‘severely sick’ demonstrate more than one of the following signs: respiratory involvement, depression, diarrhea, cyanosis of the exposed skin or wattles, edema of the face and/or head, nervous signs. The IVPI is the mean score per bird per observation over the 10-day period.

### Studies in mice

Fourteen 6- to 8-week-old (~17 g) female BALB/c mice (Vital River Laboratory, Beijing, China) from each group were anesthetized with carbon dioxide (CO_2_) and inoculated intranasally (i.n.) with 50 μl of the 10-fold dilutions of virus in phosphate-buffered saline (PBS). Each group contains five mice and the MLD_50_ value was determined by the Reed and Muench method^48^, and expressed as the TCID_50_ value corresponding to 1 MLD_50_.

Nine mice were inoculated i.n. with of 50 μl of 10^4.5^ TCID_50_ virus diluted in phosphate-buffered saline (PBS). Mock-infected control animals were inoculated i.n. with 50 μl PBS. Three mice from each group were euthanized at 3, 5, and 7 d.p.i., in which the brains and lungs were collected and homogenized using a QIAGEN TissueLyser II machine (30 cycles/s, 4 min) in 1 ml of cold PBS under sterile conditions, respectively. Solid debris was then pelleted by centrifugation at 5,000 × g for 10 min, and the homogenates were used for virus titrations in MDCK cells.

All of the mice were monitored daily for general behavior and clinical signs, including food intake, body weight, inactivity, and mortality for 14 days. Any mouse that lost ≥ 25% of its preinoculation body weight was euthanized.

### Receptor-binding assay

Viral receptor-binding specificity was determined using the solid-phase direct binding assay as described previously^49^. Briefly, 96-well microtiter plates were coated by biotinylated glycans 3’SLNLN (NeuAcα2-3Galβ1-4GlcNAcβ1-3Galβ1-4GlcNAcβ1-SpNH-LC-LC-Biotin) and 6’SLNLN (NeuAcα2-6Galβ1-4GlcNAcβ1-3Galβ1-4GlcNAcβ1-SpNH-LC-LC-Biotin), then the virus dilutions containing 128 HA units with the neuraminidase inhibitors (10 nM of each of Oseltamivir, Zanamivir, and Penrmivir) was incubated. The virus-receptor binding reaction was detected with rabbit antisera against the H5N1 virus and HRP-linked goat-anti-rabbit antibody. The results were measured by TMB (tetramethylbenzidine) at 450 nm.

### Migration routes of whooper swan and pochard

The migration routes including breeding ground, wintering ground, and the migrating directions of whooper swans (*Cygnus cygnus*) and pochards (*Aythya ferina*) in China and worldwide were mapped by the ArcGIS Desktop 10.2 software according to results of previous studies^10^,^50^,^51^,^52^.

### Ethics statement

All animal research was approved by the Chinese Academy of Sciences of Research Ethics Committee, under approval number PZIMCAS2013001, and complied with the Beijing Laboratory Animal Welfare and Ethical Guidelines of the Beijing Administration Committee of Laboratory Animals.

### Gene sequences

The GISAID accection numbers for the genome sequences of the H5N1 viruses are EPI560202-8, EPI560212, EPI560152-9, EPI559888, EPI559895, EPI559903, EPI559910, EPI559920, EPI559913, EPI559928, EPI559931, EPI559792, EPI559800, EPI559803, EPI559789, EPI559806, EPI559791, EPI559811, EPI559812, EPI559761-2, EPI559764, EPI559770-4, and EPI559724-31.

## Additional Information

**How to cite this article**: Bi, Y. *et al.* Highly Pathogenic Avian Influenza A(H5N1) Virus Struck Migratory Birds in China in 2015. *Sci. Rep.* **5**, 12986; doi: 10.1038/srep12986 (2015).

## Supplementary Material

Supplementary Information

## Figures and Tables

**Table 1:** Genetic identity (%) of the H5N1 viruses isolated from wild birds, humans, and tigers.

Virus^a^	Gene	Nucleotide (amino acid) identity (%)					
		Ws/HN/SMX1/15	Ws/HN/SMX3/15	Ws/HN/SMX4/15	Ws/HN/SMX9/15	Po/HN/SMX1/15	En/HN/SMX1/15
A/Alberta/01/2014	HA	98.8 (99.1)	98.8 (98.9)	98.8 (99.1)	98.8 (98.9)	98.7 (98.8)	98.8 (98.9)
	NA	98.8 (99.1)	98.8 (99.1)	98.8 (99.1)	98.8 (99.1)	98.8 (99.1)	98.8 (99.1)
	PB2	98.9 (98.9)	99.0 (90.0)	98.9 (98.9)	98.8 (98.5)	98.9 (99.0)	98.8 (99.0)
	PB1	99.0 (99.7)	99.0 (99.7)	99.0 (99.7)	99.0 (99.6)	99.0 (99.7)	99.0 (99.7)
	PA	99.3 (99.4)	99.4 (99.5)	99.3 (99.5)	99.4 (99.5)	99.3 (99.5)	99.4 (99.5)
	NP	99.3 (99.5)	99.1 (99.5)	99.3 (99.5)	99.2 (99.5)	99.2 (99.5)	99.2 (99.5)
	M	99.6 (M1:100, M2:100)	99.6 (M1:100, M2:100)	99.6 (M1:100, M2:100)	99.6 (M1:100, M2:100)	99.6 (M1:100, M2:100)	99.6 (M1:100, M2:100)
	NS	99.0 (NS1:98.6, NS2:99.1)	98.9 (NS1:98.2, NS2:99.1)	NS2:99.1)	98.9 (NS1:98.2, NS2:99.1)	98.9 (NS1:98.2, NS2:99.1)	98.9 (NS1:98.2, NS2:99.1)
A/tiger/Jiangsu/01/2013	HA	98.5 (98.9)	98.5 (98.7)	98.5 (98.9)	98.5 (98.7)	98.5 (98.7)	98.5 (98.7)
	NA	97.8 (98.4)	97.8 (98.4)	97.8 (98.4)	97.8 (98.4)	97.8 (98.4)	97.8 (98.4)
	PB2	91.8 (98.2)	91.8 (98.1)	91.6 (98.0)	91.6 (97.6)	91.8 (98.1)	92.0 (98.1)
	PB1	97.9 (98.9)	97.9 (98.9)	97.9 (98.9)	97.9 (98.8)	97.9 (98.9)	98.0 (98.9)
	PA	98.6 (98.7)	98.7 (98.8)	98.6 (98.8)	98.7 (98.8)	98.6 (98.8)	98.7 (98.8)
	NP	98.6 (99.5)	98.5 (99.5)	98.6 (99.5)	98.5 (99.5)	98.5 (99.5)	98.5 (99.5)
	M	95.9 (M1:100; M2:98.9)	95.9 (M1:100; M2:98.9)	95.9 (M1:100; M2:98.9)	95.9 (M1:100; M2:98.9)	95.9 (M1:100; M2:98.9)	95.9 (M1:100; M2:98.9)
	NS	97.3 (NS1:98.2; NS2:98.3)	97.2 (NS1:97.7; NS2:98.3)	97.2 (NS1:97.7; NS2:98.3)	97.2 (NS1:97.7; NS2:98.3)	97.2 (NS1:97.7; NS2:98.3)	97.2 (NS1:97.7; NS2:98.3)

^a^Ws: whooper swan; Po: pochard; En: environment; HN: Henan.

**Table 2:** Molecular characterizations of the H5N1 viruses on HA, PB2, NA, M2 and PB1-F2.

Viruses^a^	Collection date	HA (H3 numbering)						PB2			NA		M2		PB1-F2
		Connecting peptide	110	158	160	224–228	318	591	627	701	274^b^	Stalk deletion	27	31	58–90 truncated
A/Alberta/01/2014^c^	2014.1.3	RERRRKR/G	H	D	A	NRQSG	T	Q	E	D	H	49–68	I	S	Yes
A/tiger/Jiangsu/01/2013^c^	2013.1.20	RERRRKR/G	H	D	A	NGQSG	T	Q	E	D	H	49–68	I	S	Yes
Ws/HN/SMX1/2015	2015.1.4	RERRRKR/G	H	D	A	NGQSG	T	Q	E	D	H	49–68	I	S	Yes
Ws/HN/SMX3/2015	2015.1.4	RERRRKR/G	H	D	A	NGQSG	T	Q	E	D	H	49–68	I	S	Yes
Ws/HN/SMX4/2015	2015.1.5	RERRRKR/G	H	D	A	NGQSG	T	Q	E	D	H	49–68	I	S	Yes
Ws/HN/SMX9/2015	2015.1.5	RERRRKR/G	H	D	A	NGQSG	T	Q	E	D	H	49–68	I	S	Yes
Po/HN/SMX1/2015	2015.1.5	RERRRKR/G	H	D	A	NGQSG	T	Q	E	D	H	49–68	I	S	Yes
En/HN/SMX1/2015	2015.1.5	RERRRKR/G	H	D	A	NGQSG	T	Q	E	D	H	49–68	I	S	Yes

^a^Ws: whooper swan; Po: pochard; En: environment; HN: Henan.

^b^N2 numbering.

^c^The sequences of the reference strains are downloaded from GISAID and GenBank databases.

**Table 3:** Different amino acid residues in the HA of the H5N1 viruses isolated from wild birds, humans and tigers.

Virus^a^	Amino acid residue sites (H3 numbering)								
	77	193	225	246	260	397	475	509+	531
A/Alberta/01/2014	D	K	R	E	V	R	N	I	M
A/tiger/Jiangsu/01/2013	D	R	G	E	V	R	N	I	I
Ws/HN/SMX1/2015	S	K	G	E	I	K	N	V	M
Ws/HN/SMX3/2015	S	K	G	E	I	K	D	V	M
Ws/HN/SMX4/2015	S	K	G	E	I	K	N	V	M
Ws/HN/SMX9/2015	S	K	G	E	I	K	D	V	M
Po/HN/SMX1/2015	S	K	G	K	I	K	N	V	M
En/HN/SMX1/2015	S	K	G	E	I	K	D	V	M

509**+:** there is a gap after the 509^th^ residue in H3 protein compared with these H5 proteins.

^a^Ws: whooper swan; Po: pochard; En: environment; HN: Henan.

**Table 4:** Pathogenicity of Ws/Henan/SMX4/2015(H5N1) virus in chickens and mice.

Virus	TCID_50_	IVPI	MLD_50_	No. of infected mice/total no. of mice (virus titer in sample [log10 TCID_50_/ml])^a^					
				Lung			Brain		
				3	5	7	3	5	7
Ws/HN/SMX4/15	10^−7.5^/ml	2.79	10^3.0^ TCID_50_	3/3	3/3	3/3	0/3	1/3	3/3
				(2.5, 4.0, 3.5)	(4.25, 4.0, 4.75)	(2.0, 3.75, 2.5)	(—, —, —)	(—, —, <)	(1.5, 2.0, 1.5)

—, Virus was not detected in the stock homogenates. <, The virus titer was lower than 1 log10 TCID_50_.

^a^The mice were infected with 10^4.5^ TCID_50_ of virus. The brain and lung tissues of the infected mice were collected for virus titration in MDCK cells.

## References

[b1] Webster R. G., Peiris M., Chen H. & Guan Y. H5N1 outbreaks and enzootic influenza. Emerg Infect Dis 12, 3–8 (2006). 16494709 10.3201/eid1201.051024 PMC3291402

[b2] WHO. Cumulative number of confirmed human cases for avian influenza A(H5N1) reported to WHO, 2003–2015. (2015) *Available at:* http://www.who.int/influenza/human_animal_interface/H5N1_cumulative_table_archives/en/. (Accessed: 31^th^ March 2015).

[b3] WHO. Monthly Risk Assessment Summary, Influenza at the Human-Animal Interface. (2015) *Available at:* http://www.who.int/influenza/human_animal_interface/HAI_Risk_Assessment/en/. (Accessed: 31^th^ March 2015).

[b4] OIE. Update on highly pathogenic avian influenza in animals (Type H5 and H7). (2015) *Available at:* http://www.oie.int/en/animal-health-in-the-world/update-on-avian-influenza/2015/. (Accessed: 31^th^ March 2015).

[b5] Shi W. *et al.* Phylogenetics of varied subtypes of avian influenza viruses in China: potential threat to humans. Protein Cell 5, 253–257 (2014). 24622845 10.1007/s13238-014-0036-1 PMC3978160

[b6] Kim J. K., Negovetich N. J., Forrest H. L. & Webster R. G. Ducks: the “Trojan horses” of H5N1 influenza. Influenza Other Respir Viruses 3, 121–128 (2009). 19627369 10.1111/j.1750-2659.2009.00084.x PMC2749972

[b7] Olsen B. *et al.* Global patterns of influenza a virus in wild birds. Science 312, 384–388 (2006). 16627734 10.1126/science.1122438

[b8] Sturm-Ramirez K. M. *et al.* Are ducks contributing to the endemicity of highly pathogenic H5N1 influenza virus in Asia? J Virol 79, 11269–11279 (2005). 16103179 10.1128/JVI.79.17.11269-11279.2005 PMC1193583

[b9] Wang G. *et al.* H5N1 avian influenza re-emergence of Lake Qinghai: phylogenetic and antigenic analyses of the newly isolated viruses and roles of migratory birds in virus circulation. J Gen Virol 89, 697–702 (2008). 18272760 10.1099/vir.0.83419-0 PMC2885753

[b10] Tian H. *et al.* Avian influenza H5N1 viral and bird migration networks in Asia. Proc Natl Acad Sci USA 112, 172–177 (2015). 25535385 10.1073/pnas.1405216112 PMC4291667

[b11] Li X. *et al.* Global and local persistence of influenza A(H5N1) virus. Emerg Infect Dis 20, 1287–1295 (2014). 25061965 10.3201/eid2008.130910 PMC4111173

[b12] Liu J. *et al.* Highly pathogenic H5N1 influenza virus infection in migratory birds. Science 309, 1206 (2005). 16000410 10.1126/science.1115273

[b13] Kwon H. I. *et al.* Genetic characterization and pathogenicity assessment of highly pathogenic H5N1 avian influenza viruses isolated from migratory wild birds in 2011, South Korea. Virus Res 160, 305–315 (2011). 21782862 10.1016/j.virusres.2011.07.003

[b14] Watanabe Y. *et al.* Acquisition of human-type receptor binding specificity by new H5N1 influenza virus sublineages during their emergence in birds in Egypt. PLoS Pathog 7, e1002068 (2011). 21637809 10.1371/journal.ppat.1002068 PMC3102706

[b15] Saad M. D. *et al.* Possible avian influenza (H5N1) from migratory bird, Egypt. Emerg Infect Dis 13, 1120–1121 (2007). 18214200 10.3201/eid1307.061222 PMC2878221

[b16] Hu X. *et al.* Clade 2.3.2 avian influenza virus (H5N1), Qinghai Lake region, China, 2009–2010. Emerg Infect Dis 17, 560–562 (2011). 21392463 10.3201/eid1703.100948 PMC3166005

[b17] Li Y. *et al.* New avian influenza virus (H5N1) in wild birds, Qinghai, China. Emerg Infect Dis 17, 265–267 (2011). 21291602 10.3201/eid1702.100732 PMC3204760

[b18] Sakoda Y. *et al.* Characterization of H5N1 highly pathogenic avian influenza virus strains isolated from migratory waterfowl in Mongolia on the way back from the southern Asia to their northern territory. Virology 406, 88–94 (2010). 20673942 10.1016/j.virol.2010.07.007

[b19] Sakoda Y. *et al.* Reintroduction of H5N1 highly pathogenic avian influenza virus by migratory water birds, causing poultry outbreaks in the 2010-2011 winter season in Japan. J Gen Virol 93, 541–550 (2012). 22113008 10.1099/vir.0.037572-0

[b20] Pabbaraju K. *et al.* Full-genome analysis of avian influenza A(H5N1) virus from a human, North America, 2013. Emerg Infect Dis 20, 887–891 (2014). 24755439 10.3201/eid2005.140164 PMC4012823

[b21] He S. *et al.* Lethal infection by a novel reassortant H5N1 avian influenza A virus in a zoo-housed tiger. Microbes Infect 17, 54–61 (2015). 25461468 10.1016/j.micinf.2014.10.004

[b22] Zhang Y. *et al.* A Single Amino Acid at the Hemagglutinin Cleavage Site Contributes to the Pathogenicity and Neurovirulence of H5N1 Influenza Virus in Mice. Journal of Virology 86, 6924–6931 (2012). 22496231 10.1128/JVI.07142-11 PMC3393526

[b23] Zhang W. *et al.* An airborne transmissible avian influenza H5 hemagglutinin seen at the atomic level. Science 340, 1463–1467 (2013). 23641058 10.1126/science.1236787

[b24] Shi Y., Wu Y., Zhang W., Qi J. & Gao G. F. Enabling the ‘host jump’: structural determinants of receptor-binding specificity in influenza A viruses. Nat Rev Microbiol 12, 822–831 (2014). 25383601 10.1038/nrmicro3362

[b25] Lu X. *et al.* Structure and receptor-binding properties of an airborne transmissible avian influenza A virus hemagglutinin H5 (VN1203mut). Protein Cell 4, 502–511 (2013). 23794001 10.1007/s13238-013-3906-z PMC4875516

[b26] Maurer-Stroh S. *et al.* Potential human adaptation mutation of influenza A(H5N1) virus, Canada. Emerg Infect Dis 20, 1580–1582 (2014). 25153690 10.3201/eid2009.140240 PMC4178386

[b27] Matsuoka Y. *et al.* Neuraminidase stalk length and additional glycosylation of the hemagglutinin influence the virulence of influenza H5N1 viruses for mice. J Virol 83, 4704–4708 (2009). 19225004 10.1128/JVI.01987-08 PMC2668507

[b28] Zhou H. *et al.* The special neuraminidase stalk-motif responsible for increased virulence and pathogenesis of H5N1 influenza A virus. PLoS One 4, e6277 (2009). 19609439 10.1371/journal.pone.0006277 PMC2707603

[b29] McAuley J. L. *et al.* PB1-F2 proteins from H5N1 and 20 century pandemic influenza viruses cause immunopathology. PLoS Pathog 6, e1001014 (2010). 20661425 10.1371/journal.ppat.1001014 PMC2908617

[b30] Zamarin D., Ortigoza M. B. & Palese P. Influenza A virus PB1-F2 protein contributes to viral pathogenesis in mice. J Virol 80, 7976–7983 (2006). 16873254 10.1128/JVI.00415-06 PMC1563817

[b31] Keawcharoen J. *et al.* Wild ducks as long-distance vectors of highly pathogenic avian influenza virus (H5N1). Emerg Infect Dis 14, 600–607 (2008). 18394278 10.3201/eid1404.071016 PMC2570914

[b32] Uchida Y. *et al.* Highly pathogenic avian influenza virus (H5N1) isolated from whooper swans, Japan. Emerg Infect Dis 14, 1427–1429 (2008). 18760011 10.3201/eid1409.080655 PMC2603097

[b33] China whooper swan research center. (2015) *Available at:* http://www.datiane.com.cn/. (Accessed: 16^th^ April 2015).

[b34] Fan S. *et al.* A novel highly pathogenic H5N8 avian influenza virus isolated from a wild duck in China. Influenza Other Respir Viruses 8, 646–653 (2014). 25363159 10.1111/irv.12289 PMC4262280

[b35] Jeong J. *et al.* Highly pathogenic avian influenza virus (H5N8) in domestic poultry and its relationship with migratory birds in South Korea during 2014. Vet Microbiol 173, 249–257 (2014). 25192767 10.1016/j.vetmic.2014.08.002

[b36] Food and Agriculture Organization (FAO). Avian influenza A(H5N8) detected in Europe…a journey to the West? (2014) *Available at:* http://www.fao.org/ag/againfo/home/en/news_archive/2014_A-H5N8_detected_in_Europe.html, (Accessed: 14^th^ November 2014).

[b37] Lee D. H. *et al.* Intercontinental Spread of Asian-origin H5N8 to North America through Beringia by Migratory Birds. J Virol 89, 6521–6524 (2015). 25855748 10.1128/JVI.00728-15 PMC4474297

[b38] Pasick J. *et al.* Reassortant Highly Pathogenic Influenza A H5N2 Virus Containing Gene Segments Related to Eurasian H5N8 in British Columbia, Canada, 2014. Sci Rep 5, 9484 (2015). 25804829 10.1038/srep09484 PMC4372658

[b39] Verhagen J. H., Herfst S. & Fouchier R. A. M. How a virus travels the world. Science 347, 616–617 (2015). 25657235 10.1126/science.aaa6724

[b40] Yingst S. L., Saad M. D. & Felt S. A. Q. inghai-like H5N1 from domestic cats, northern Iraq. Emerg Infect Dis 12, 1295–1297 (2006). 16972356 10.3201/eid1208.060264 PMC3291230

[b41] Abdel-Moneim A. S., Abdel-Ghany A. E. & Shany S. A. Isolation and characterization of highly pathogenic avian influenza virus subtype H5N1 from donkeys. J Biomed Sci 17, 25 (2010). 20398268 10.1186/1423-0127-17-25 PMC2867947

[b42] WHO Global Influenza Surveillance Network. Manual for the laboratory diagnosis and virological surveillance of influenza. Part 2.E. (2011). *Available at:* http://www.who.int/influenza/gisrs_laboratory/manual_diagnosis_surveillance_influenza/en/. (Accessed: 11^th^ June 2014).

[b43] Bi Y. *et al.* Two Novel Reassortants of Avian Influenza A(H5N6) Virus in China. J Gen Virol 96, 975–981 (2015). 25604926 10.1099/vir.0.000056

[b44] Edgar R. C. MUSCLE: multiple sequence alignment with high accuracy and high throughput. Nucleic Acids Res 32, 1792–1797 (2004). 15034147 10.1093/nar/gkh340 PMC390337

[b45] Stamatakis A. RAxML version 8: a tool for phylogenetic analysis and post-analysis of large phylogenies. Bioinformatics 30, 1312–1313 (2014). 24451623 10.1093/bioinformatics/btu033 PMC3998144

[b46] Bi Y. H. *et al.* Novel genetic reassortants in H9N2 influenza A viruses and their diverse pathogenicity to mice. Virol J 8, 505 (2011). 22050764 10.1186/1743-422X-8-505 PMC3236014

[b47] Manual of Diagnostic Tests and Vaccines for Terrestrial Animals 2014. Chapter 2.3.4. *Available at:* http://www.oie.int/en/international-standard-setting/terrestrial-manual/access-online/(2014). (Accessed: 22^th^ January 2014).

[b48] Reed L. J. & Muench H. A simple method of estimating fifty percent endpoints. The American Journal of Hygiene. 27, 493–497. (1938).

[b49] Shi Y. *et al.* Structures and receptor binding of hemagglutinins from human-infecting H7N9 influenza viruses. Science 342, 243–247 (2013). 24009358 10.1126/science.1242917

[b50] BirdLife International and NatureServe (2014) Bird species distribution maps of the world. BirdLife International, Cambridge, UK and NatureServe, Arlington, USA.

[b51] Del Hoyo J., Elliott A., Sargatal J., Christie D. A. & de Juana E. (eds.). BirdLife International and NatureServe (2014) Bird species distribution maps of the world. Handbook of the Birds of the World Alive: Vol 1: Ostrich to Duck. Lynx Edicions, Barcelona. (2013).

[b52] MacKinnon J., Phillipps K., He F. Q., Showler D. & MacKinnon D. A field guide to the birds of China. Oxford, United Kingdom, Oxford University Press. (2000).

