# Supplementary material for: Highly Pathogenic Avian Influenza A(H5N1) Virus Struck Migratory Birds in China in 2015
Source: Sci Rep. 2015 Aug 11;5:12986. doi: 10.1038/srep12986 (PMC4531313; doi:10.1038/srep12986)
Supplement: Supplementary Information [file srep12986-s1.pdf]

Supplementary Information

Title: Highly Pathogenic Avian Influenza A(H5N1) Virus Struck Migratory Birds in China in 2015

Authors: Yuhai Bi, Zhenjie Zhang, Wenjun Liu, Yanbo Yin, Jianmin Hong, Xiangdong Li, Haiming Wang, Gary Wong, Jianjun Chen, Yunfeng Li, Wendong Ru, Ruyi Gao, Di Liu, Yingxia Liu, Boping Zhou, George F. Gao, Weifeng Shi, Fumin Lei

Supplementary Figure S1-8

The reference sequences were downloaded from the GISAID and GenBank database. All of the phylogenetic analyses were performed using Raxml, with 1000 bootstrap replicates. The HA (Fig.S1), NA (Fig.S2), NP (Fig.S3), M (Fig.S4), NS (Fig.S5), PA (Fig.S6), PB1 (Fig.S7), PB2 (Fig.S8) segments of the whooper swan-H5N1, A/Alberta/01/2014(H5N1), and A/tiger/Jiangsu/01/2013(H5N1) viruses are colored in red, pink and green, respectively.

Fig.S1

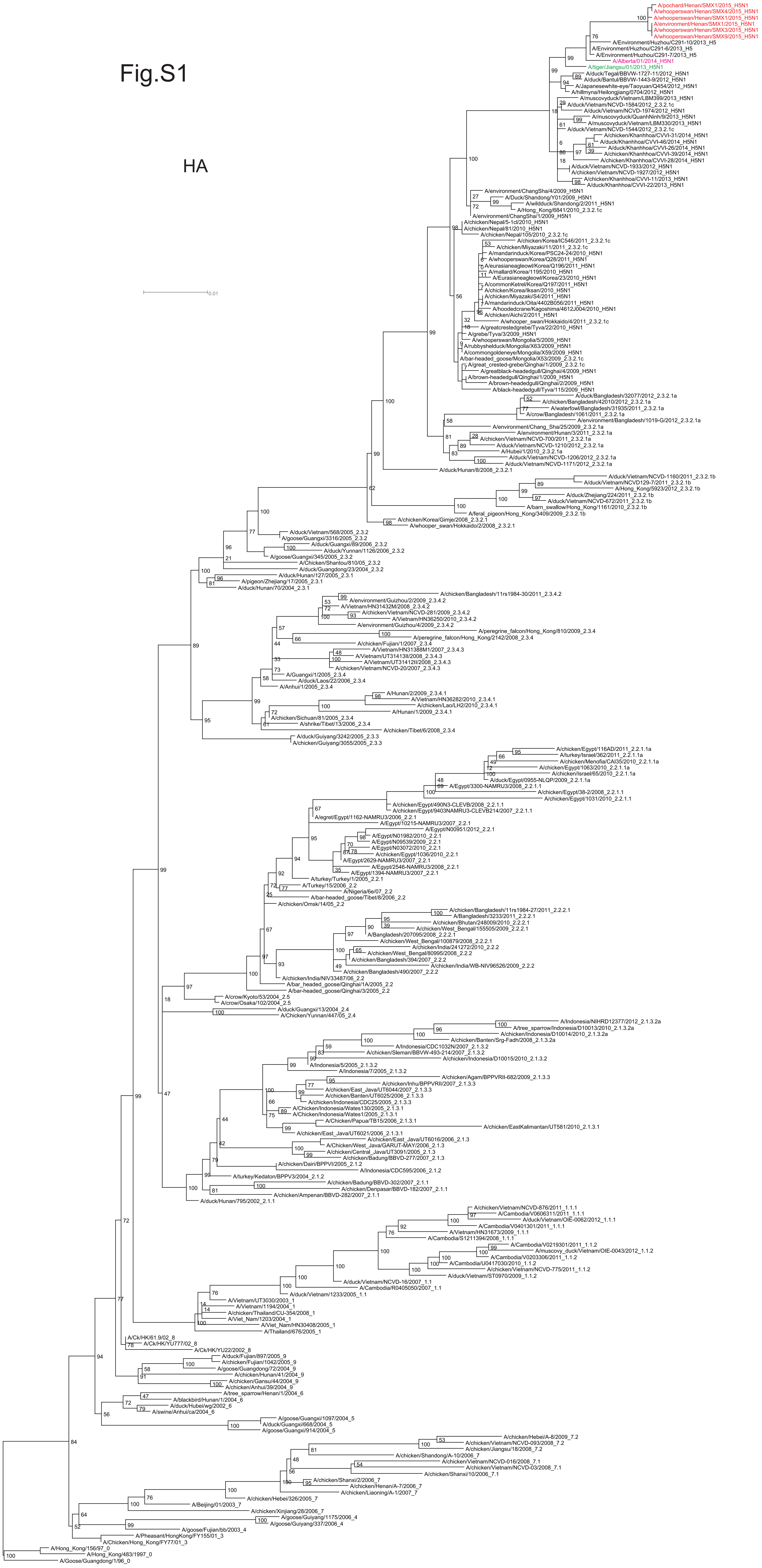

Fig.S2

NA

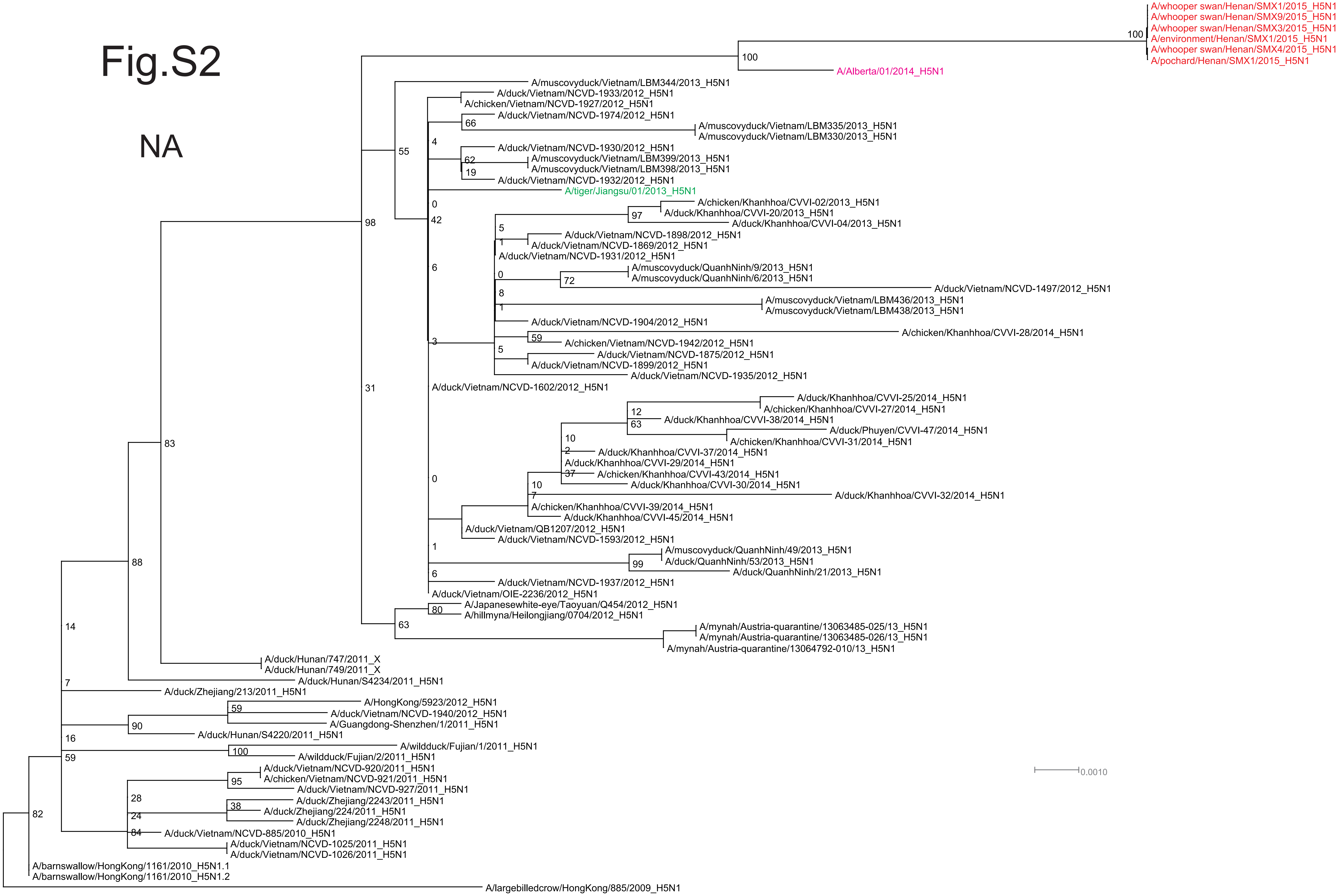
A/whooper swan/Henan/SMX1/2015_H5N1
A/whooper swan/Henan/SMX9/2015_H5N1
A/whooper swan/Henan/SMX3/2015_H5N1
A/environment/Henan/SMX1/2015_H5N1
A/whooper swan/Henan/SMX4/2015_H5N1
A/pochard/Henan/SMX1/2015_H5N1
A/Alberta/01/2014_H5N1
A/muscovyduck/Vietnam/LBM344/2013_H5N1
A/duck/Vietnam/NCVD-1933/2012_H5N1
A/chicken/Vietnam/NCVD-1927/2012_H5N1
A/duck/Vietnam/NCVD-1974/2012_H5N1
A/muscovyduck/Vietnam/LBM335/2013_H5N1
A/muscovyduck/Vietnam/LBM330/2013_H5N1
A/duck/Vietnam/NCVD-1930/2012_H5N1
A/muscovyduck/Vietnam/LBM399/2013_H5N1
A/muscovyduck/Vietnam/LBM398/2013_H5N1
A/duck/Vietnam/NCVD-1932/2012_H5N1
A/tiger/Jiangsu/01/2013_H5N1
A/chicken/Khanhhoa/CVVI-02/2013_H5N1
A/duck/Khanhhoa/CVVI-20/2013_H5N1
A/duck/Khanhhoa/CVVI-04/2013_H5N1
A/duck/Vietnam/NCVD-1898/2012_H5N1
A/duck/Vietnam/NCVD-1869/2012_H5N1
A/duck/Vietnam/NCVD-1931/2012_H5N1
A/muscovyduck/QuanhNinh/9/2013_H5N1
A/muscovyduck/QuanhNinh/6/2013_H5N1
A/duck/Vietnam/NCVD-1497/2012_H5N1
A/muscovyduck/Vietnam/LBM436/2013_H5N1
A/muscovyduck/Vietnam/LBM438/2013_H5N1
A/duck/Vietnam/NCVD-1904/2012_H5N1
A/chicken/Khanhhoa/CVVI-28/2014_H5N1
A/chicken/Vietnam/NCVD-1942/2012_H5N1
A/duck/Vietnam/NCVD-1875/2012_H5N1
A/duck/Vietnam/NCVD-1899/2012_H5N1
A/duck/Vietnam/NCVD-1935/2012_H5N1
A/duck/Vietnam/NCVD-1602/2012_H5N1
A/duck/Khanhhoa/CVVI-25/2014_H5N1
A/chicken/Khanhhoa/CVVI-27/2014_H5N1
A/duck/Khanhhoa/CVVI-38/2014_H5N1
A/duck/Phuyen/CVVI-47/2014_H5N1
A/chicken/Khanhhoa/CVVI-31/2014_H5N1
A/duck/Khanhhoa/CVVI-37/2014_H5N1
A/duck/Khanhhoa/CVVI-29/2014_H5N1
A/chicken/Khanhhoa/CVVI-43/2014_H5N1
A/duck/Khanhhoa/CVVI-30/2014_H5N1
A/duck/Khanhhoa/CVVI-32/2014_H5N1
A/chicken/Khanhhoa/CVVI-39/2014_H5N1
A/duck/Khanhhoa/CVVI-45/2014_H5N1
A/duck/Vietnam/QB1207/2012_H5N1
A/duck/Vietnam/NCVD-1593/2012_H5N1
A/muscovyduck/QuanhNinh/49/2013_H5N1
A/duck/QuanhNinh/53/2013_H5N1
A/duck/QuanhNinh/21/2013_H5N1
A/duck/Vietnam/NCVD-1937/2012_H5N1
A/duck/Vietnam/OIE-2236/2012_H5N1
A/Japanesewhite-eye/Taoyuan/Q454/2012_H5N1
A/hillmyna/Heilongjiang/0704/2012_H5N1
A/mynah/Austria-quarantine/13063485-025/13_H5N1
A/mynah/Austria-quarantine/13063485-026/13_H5N1
A/mynah/Austria-quarantine/13064792-010/13_H5N1
A/duck/Hunan/747/2011_X
A/duck/Hunan/749/2011_X
A/duck/Hunan/S4234/2011_H5N1
A/duck/Zhejiang/213/2011_H5N1
A/HongKong/5923/2012_H5N1
A/duck/Vietnam/NCVD-1940/2012_H5N1
A/Guangdong-Shenzhen/1/2011_H5N1
A/duck/Hunan/S4220/2011_H5N1
A/wildduck/Fujian/1/2011_H5N1
A/wildduck/Fujian/2/2011_H5N1
A/duck/Vietnam/NCVD-920/2011_H5N1
A/chicken/Vietnam/NCVD-921/2011_H5N1
A/duck/Vietnam/NCVD-927/2011_H5N1
A/duck/Zhejiang/2243/2011_H5N1
A/duck/Zhejiang/224/2011_H5N1
A/duck/Zhejiang/2248/2011_H5N1
A/duck/Vietnam/NCVD-885/2010_H5N1
A/duck/Vietnam/NCVD-1025/2011_H5N1
A/duck/Vietnam/NCVD-1026/2011_H5N1
A/barnswallow/HongKong/1161/2010_H5N1.1
A/barnswallow/HongKong/1161/2010_H5N1.2
A/largebilledcrow/HongKong/885/2009_H5N1
0.0010

Fig.S3

NP

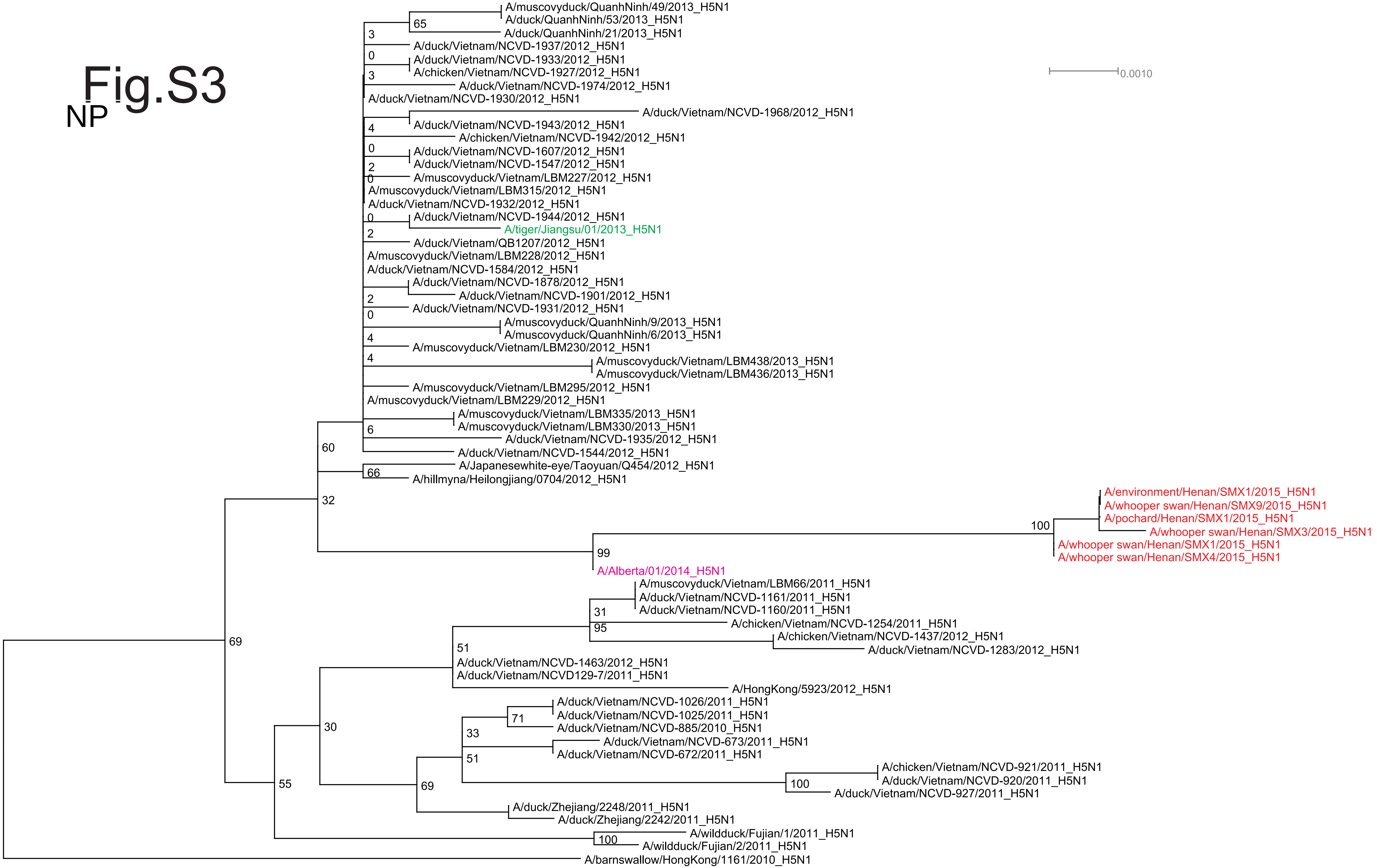
0.0010
A/muscovyduck/QuanhNinh/49/2013_H5N1
A/duck/QuanhNinh/53/2013_H5N1
A/duck/QuanhNinh/21/2013_H5N1
A/duck/Vietnam/NCVD-1937/2012_H5N1
A/duck/Vietnam/NCVD-1933/2012_H5N1
A/chicken/Vietnam/NCVD-1927/2012_H5N1
A/duck/Vietnam/NCVD-1974/2012_H5N1
A/duck/Vietnam/NCVD-1930/2012_H5N1
A/duck/Vietnam/NCVD-1968/2012_H5N1
A/duck/Vietnam/NCVD-1943/2012_H5N1
A/chicken/Vietnam/NCVD-1942/2012_H5N1
A/duck/Vietnam/NCVD-1607/2012_H5N1
A/duck/Vietnam/NCVD-1547/2012_H5N1
A/muscovyduck/Vietnam/LBM227/2012_H5N1
A/muscovyduck/Vietnam/LBM315/2012_H5N1
A/duck/Vietnam/NCVD-1932/2012_H5N1
A/duck/Vietnam/NCVD-1944/2012_H5N1
A/tiger/Jiangsu/01/2013_H5N1
A/duck/Vietnam/QB1207/2012_H5N1
A/muscovyduck/Vietnam/LBM228/2012_H5N1
A/duck/Vietnam/NCVD-1584/2012_H5N1
A/duck/Vietnam/NCVD-1878/2012_H5N1
A/duck/Vietnam/NCVD-1901/2012_H5N1
A/duck/Vietnam/NCVD-1931/2012_H5N1
A/muscovyduck/QuanhNinh/9/2013_H5N1
A/muscovyduck/QuanhNinh/6/2013_H5N1
A/muscovyduck/Vietnam/LBM230/2012_H5N1
A/muscovyduck/Vietnam/LBM438/2013_H5N1
A/muscovyduck/Vietnam/LBM436/2013_H5N1
A/muscovyduck/Vietnam/LBM295/2012_H5N1
A/muscovyduck/Vietnam/LBM229/2012_H5N1
A/muscovyduck/Vietnam/LBM335/2013_H5N1
A/muscovyduck/Vietnam/LBM330/2013_H5N1
A/duck/Vietnam/NCVD-1935/2012_H5N1
A/duck/Vietnam/NCVD-1544/2012_H5N1
A/Japanesewhite-eye/Taoyuan/Q454/2012_H5N1
A/hillmyna/Heilongjiang/0704/2012_H5N1
A/environment/Henan/SMX1/2015_H5N1
A/whooper swan/Henan/SMX9/2015_H5N1
A/pochard/Henan/SMX1/2015_H5N1
A/whooper swan/Henan/SMX3/2015_H5N1
A/whooper swan/Henan/SMX1/2015_H5N1
A/whooper swan/Henan/SMX4/2015_H5N1
A/Alberta/01/2014_H5N1
A/muscovyduck/Vietnam/LBM66/2011_H5N1
A/duck/Vietnam/NCVD-1161/2011_H5N1
A/duck/Vietnam/NCVD-1160/2011_H5N1
A/chicken/Vietnam/NCVD-1254/2011_H5N1
A/chicken/Vietnam/NCVD-1437/2012_H5N1
A/duck/Vietnam/NCVD-1283/2012_H5N1
A/duck/Vietnam/NCVD-1463/2012_H5N1
A/duck/Vietnam/NCVD129-7/2011_H5N1
A/HongKong/5923/2012_H5N1
A/duck/Vietnam/NCVD-1026/2011_H5N1
A/duck/Vietnam/NCVD-1025/2011_H5N1
A/duck/Vietnam/NCVD-885/2010_H5N1
A/duck/Vietnam/NCVD-673/2011_H5N1
A/duck/Vietnam/NCVD-672/2011_H5N1
A/chicken/Vietnam/NCVD-921/2011_H5N1
A/duck/Vietnam/NCVD-920/2011_H5N1
A/duck/Vietnam/NCVD-927/2011_H5N1
A/duck/Zhejiang/2248/2011_H5N1
A/duck/Zhejiang/2242/2011_H5N1
A/wildduck/Fujian/1/2011_H5N1
A/wildduck/Fujian/2/2011_H5N1
A/barnswallow/HongKong/1161/2010_H5N1

Fig.S4

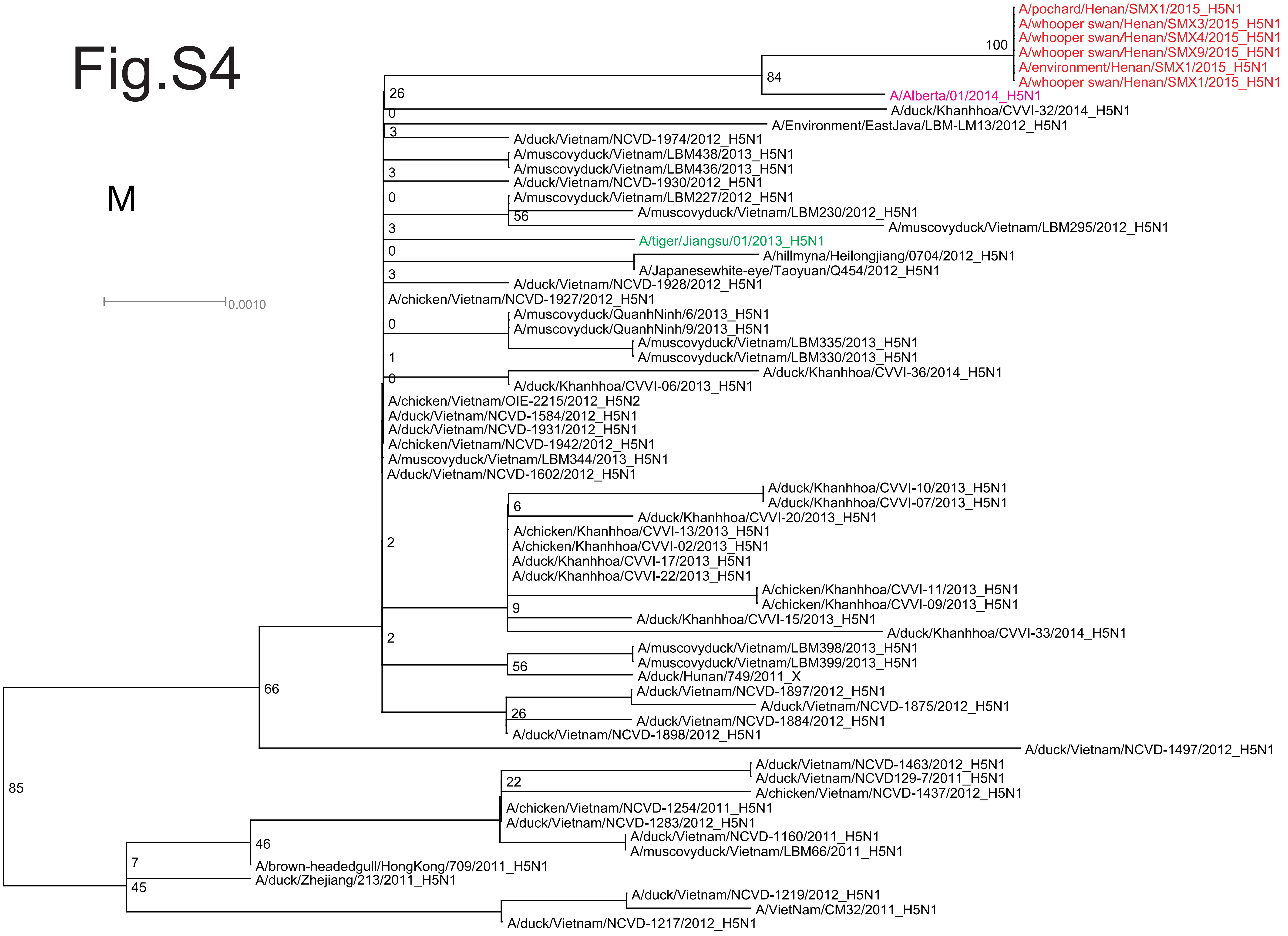
M
0.0010
A/pochard/Henan/SMX1/2015_H5N1
A/whooper swan/Henan/SMX3/2015_H5N1
A/whooper swan/Henan/SMX4/2015_H5N1
A/whooper swan/Henan/SMX9/2015_H5N1
A/environment/Henan/SMX1/2015_H5N1
A/whooper swan/Henan/SMX1/2015_H5N1
100
84
26
A/Alberta/01/2014_H5N1
0
A/duck/Khanhhoa/CVVI-32/2014_H5N1
A/Environment/EastJava/LBM-LM13/2012_H5N1
3
A/duck/Vietnam/NCVD-1974/2012_H5N1
A/muscovyduck/Vietnam/LBM438/2013_H5N1
3
A/muscovyduck/Vietnam/LBM436/2013_H5N1
A/duck/Vietnam/NCVD-1930/2012_H5N1
0
A/muscovyduck/Vietnam/LBM227/2012_H5N1
56
A/muscovyduck/Vietnam/LBM230/2012_H5N1
3
A/muscovyduck/Vietnam/LBM295/2012_H5N1
A/tiger/Jiangsu/01/2013_H5N1
0
A/hillmyna/Heilongjiang/0704/2012_H5N1
A/Japanesewhite-eye/Taoyuan/Q454/2012_H5N1
3
A/duck/Vietnam/NCVD-1928/2012_H5N1
A/chicken/Vietnam/NCVD-1927/2012_H5N1
A/muscovyduck/QuanhNinh/6/2013_H5N1
0
A/muscovyduck/QuanhNinh/9/2013_H5N1
A/muscovyduck/Vietnam/LBM335/2013_H5N1
1
A/muscovyduck/Vietnam/LBM330/2013_H5N1
0
A/duck/Khanhhoa/CVVI-36/2014_H5N1
A/duck/Khanhhoa/CVVI-06/2013_H5N1
A/chicken/Vietnam/OIE-2215/2012_H5N2
A/duck/Vietnam/NCVD-1584/2012_H5N1
A/duck/Vietnam/NCVD-1931/2012_H5N1
A/chicken/Vietnam/NCVD-1942/2012_H5N1
A/muscovyduck/Vietnam/LBM344/2013_H5N1
A/duck/Vietnam/NCVD-1602/2012_H5N1
A/duck/Khanhhoa/CVVI-10/2013_H5N1
6
A/duck/Khanhhoa/CVVI-07/2013_H5N1
A/duck/Khanhhoa/CVVI-20/2013_H5N1
A/chicken/Khanhhoa/CVVI-13/2013_H5N1
A/chicken/Khanhhoa/CVVI-02/2013_H5N1
2
A/duck/Khanhhoa/CVVI-17/2013_H5N1
A/duck/Khanhhoa/CVVI-22/2013_H5N1
A/chicken/Khanhhoa/CVVI-11/2013_H5N1
A/chicken/Khanhhoa/CVVI-09/2013_H5N1
9
A/duck/Khanhhoa/CVVI-15/2013_H5N1
A/duck/Khanhhoa/CVVI-33/2014_H5N1
2
A/muscovyduck/Vietnam/LBM398/2013_H5N1
56
A/muscovyduck/Vietnam/LBM399/2013_H5N1
A/duck/Hunan/749/2011_X
66
A/duck/Vietnam/NCVD-1897/2012_H5N1
A/duck/Vietnam/NCVD-1875/2012_H5N1
26
A/duck/Vietnam/NCVD-1884/2012_H5N1
A/duck/Vietnam/NCVD-1898/2012_H5N1
A/duck/Vietnam/NCVD-1497/2012_H5N1
A/duck/Vietnam/NCVD-1463/2012_H5N1
A/duck/Vietnam/NCVD129-7/2011_H5N1
85
22
A/chicken/Vietnam/NCVD-1437/2012_H5N1
A/chicken/Vietnam/NCVD-1254/2011_H5N1
A/duck/Vietnam/NCVD-1283/2012_H5N1
A/duck/Vietnam/NCVD-1160/2011_H5N1
46
A/muscovyduck/Vietnam/LBM66/2011_H5N1
7
A/brown-headedgull/HongKong/709/2011_H5N1
45
A/duck/Zhejiang/213/2011_H5N1
A/duck/Vietnam/NCVD-1219/2012_H5N1
A/VietNam/CM32/2011_H5N1
A/duck/Vietnam/NCVD-1217/2012_H5N1

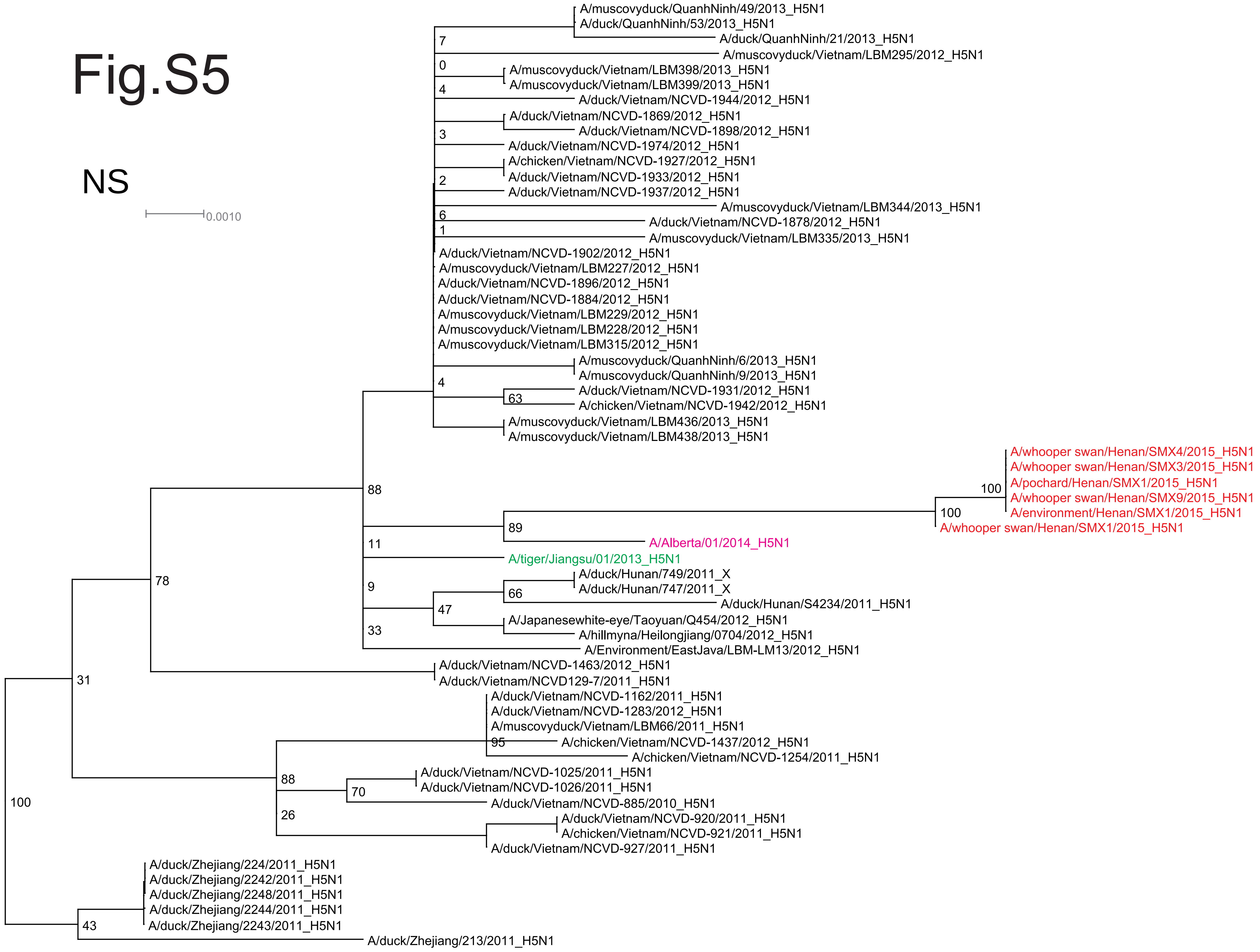
NS
0.0010
A/muscovyduck/QuanhNinh/49/2013_H5N1
A/duck/QuanhNinh/53/2013_H5N1
A/duck/QuanhNinh/21/2013_H5N1
A/muscovyduck/Vietnam/LBM295/2012_H5N1
A/muscovyduck/Vietnam/LBM398/2013_H5N1
A/muscovyduck/Vietnam/LBM399/2013_H5N1
A/duck/Vietnam/NCVD-1944/2012_H5N1
A/duck/Vietnam/NCVD-1869/2012_H5N1
A/duck/Vietnam/NCVD-1898/2012_H5N1
A/duck/Vietnam/NCVD-1974/2012_H5N1
A/chicken/Vietnam/NCVD-1927/2012_H5N1
A/duck/Vietnam/NCVD-1933/2012_H5N1
A/duck/Vietnam/NCVD-1937/2012_H5N1
A/muscovyduck/Vietnam/LBM344/2013_H5N1
A/duck/Vietnam/NCVD-1878/2012_H5N1
A/muscovyduck/Vietnam/LBM335/2013_H5N1
A/duck/Vietnam/NCVD-1902/2012_H5N1
A/muscovyduck/Vietnam/LBM227/2012_H5N1
A/duck/Vietnam/NCVD-1896/2012_H5N1
A/duck/Vietnam/NCVD-1884/2012_H5N1
A/muscovyduck/Vietnam/LBM229/2012_H5N1
A/muscovyduck/Vietnam/LBM228/2012_H5N1
A/muscovyduck/Vietnam/LBM315/2012_H5N1
A/muscovyduck/QuanhNinh/6/2013_H5N1
A/muscovyduck/QuanhNinh/9/2013_H5N1
A/duck/Vietnam/NCVD-1931/2012_H5N1
A/chicken/Vietnam/NCVD-1942/2012_H5N1
A/muscovyduck/Vietnam/LBM436/2013_H5N1
A/muscovyduck/Vietnam/LBM438/2013_H5N1
A/whooper swan/Henan/SMX4/2015_H5N1
A/whooper swan/Henan/SMX3/2015_H5N1
A/pochard/Henan/SMX1/2015_H5N1
A/whooper swan/Henan/SMX9/2015_H5N1
A/environment/Henan/SMX1/2015_H5N1
A/whooper swan/Henan/SMX1/2015_H5N1
A/Alberta/01/2014_H5N1
A/tiger/Jiangsu/01/2013_H5N1
A/duck/Hunan/749/2011_X
A/duck/Hunan/747/2011_X
A/duck/Hunan/S4234/2011_H5N1
A/Japanesewhite-eye/Taoyuan/Q454/2012_H5N1
A/hillmyna/Heilongjiang/0704/2012_H5N1
A/Environment/EastJava/LBM-LM13/2012_H5N1
A/duck/Vietnam/NCVD-1463/2012_H5N1
A/duck/Vietnam/NCVD129-7/2011_H5N1
A/duck/Vietnam/NCVD-1162/2011_H5N1
A/duck/Vietnam/NCVD-1283/2012_H5N1
A/muscovyduck/Vietnam/LBM66/2011_H5N1
A/chicken/Vietnam/NCVD-1437/2012_H5N1
A/chicken/Vietnam/NCVD-1254/2011_H5N1
A/duck/Vietnam/NCVD-1025/2011_H5N1
A/duck/Vietnam/NCVD-1026/2011_H5N1
A/duck/Vietnam/NCVD-885/2010_H5N1
A/duck/Vietnam/NCVD-920/2011_H5N1
A/chicken/Vietnam/NCVD-921/2011_H5N1
A/duck/Vietnam/NCVD-927/2011_H5N1
A/duck/Zhejiang/224/2011_H5N1
A/duck/Zhejiang/2242/2011_H5N1
A/duck/Zhejiang/2248/2011_H5N1
A/duck/Zhejiang/2244/2011_H5N1
A/duck/Zhejiang/2243/2011_H5N1
A/duck/Zhejiang/213/2011_H5N1

Fig.S5

Fig.S6

PA

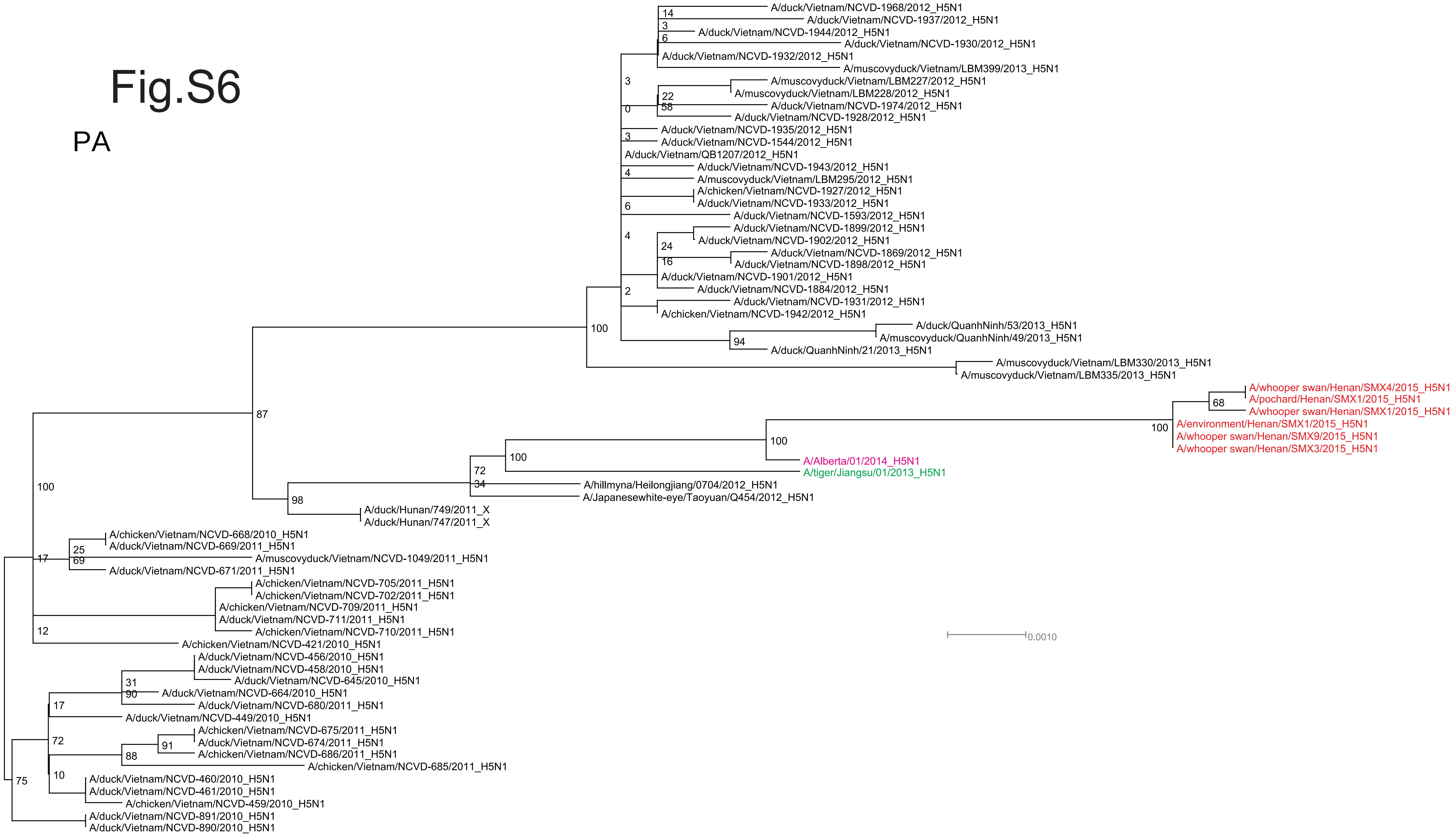
A/duck/Vietnam/NCVD-1968/2012_H5N1
A/duck/Vietnam/NCVD-1937/2012_H5N1
A/duck/Vietnam/NCVD-1944/2012_H5N1
A/duck/Vietnam/NCVD-1930/2012_H5N1
A/duck/Vietnam/NCVD-1932/2012_H5N1
A/muscovyduck/Vietnam/LBM399/2013_H5N1
A/muscovyduck/Vietnam/LBM227/2012_H5N1
A/muscovyduck/Vietnam/LBM228/2012_H5N1
A/duck/Vietnam/NCVD-1974/2012_H5N1
A/duck/Vietnam/NCVD-1928/2012_H5N1
A/duck/Vietnam/NCVD-1935/2012_H5N1
A/duck/Vietnam/NCVD-1544/2012_H5N1
A/duck/Vietnam/QB1207/2012_H5N1
A/duck/Vietnam/NCVD-1943/2012_H5N1
A/muscovyduck/Vietnam/LBM295/2012_H5N1
A/chicken/Vietnam/NCVD-1927/2012_H5N1
A/duck/Vietnam/NCVD-1933/2012_H5N1
A/duck/Vietnam/NCVD-1593/2012_H5N1
A/duck/Vietnam/NCVD-1899/2012_H5N1
A/duck/Vietnam/NCVD-1902/2012_H5N1
A/duck/Vietnam/NCVD-1869/2012_H5N1
A/duck/Vietnam/NCVD-1898/2012_H5N1
A/duck/Vietnam/NCVD-1901/2012_H5N1
A/duck/Vietnam/NCVD-1884/2012_H5N1
A/duck/Vietnam/NCVD-1931/2012_H5N1
A/chicken/Vietnam/NCVD-1942/2012_H5N1
A/duck/QuanhNinh/53/2013_H5N1
A/muscovyduck/QuanhNinh/49/2013_H5N1
A/duck/QuanhNinh/21/2013_H5N1
A/muscovyduck/Vietnam/LBM330/2013_H5N1
A/muscovyduck/Vietnam/LBM335/2013_H5N1
A/whooper swan/Henan/SMX4/2015_H5N1
A/pochard/Henan/SMX1/2015_H5N1
A/whooper swan/Henan/SMX1/2015_H5N1
A/environment/Henan/SMX1/2015_H5N1
A/whooper swan/Henan/SMX9/2015_H5N1
A/whooper swan/Henan/SMX3/2015_H5N1
A/Alberta/01/2014_H5N1
A/tiger/Jiangsu/01/2013_H5N1
A/hillmyna/Heilongjiang/0704/2012_H5N1
A/Japanesewhite-eye/Taoyuan/Q454/2012_H5N1
A/duck/Hunan/749/2011_X
A/duck/Hunan/747/2011_X
A/chicken/Vietnam/NCVD-668/2010_H5N1
A/duck/Vietnam/NCVD-669/2011_H5N1
A/muscovyduck/Vietnam/NCVD-1049/2011_H5N1
A/duck/Vietnam/NCVD-671/2011_H5N1
A/chicken/Vietnam/NCVD-705/2011_H5N1
A/chicken/Vietnam/NCVD-702/2011_H5N1
A/chicken/Vietnam/NCVD-709/2011_H5N1
A/duck/Vietnam/NCVD-711/2011_H5N1
A/chicken/Vietnam/NCVD-710/2011_H5N1
A/chicken/Vietnam/NCVD-421/2010_H5N1
A/duck/Vietnam/NCVD-456/2010_H5N1
A/duck/Vietnam/NCVD-458/2010_H5N1
A/duck/Vietnam/NCVD-645/2010_H5N1
A/duck/Vietnam/NCVD-664/2010_H5N1
A/duck/Vietnam/NCVD-680/2011_H5N1
A/duck/Vietnam/NCVD-449/2010_H5N1
A/chicken/Vietnam/NCVD-675/2011_H5N1
A/duck/Vietnam/NCVD-674/2011_H5N1
A/chicken/Vietnam/NCVD-686/2011_H5N1
A/chicken/Vietnam/NCVD-685/2011_H5N1
A/duck/Vietnam/NCVD-460/2010_H5N1
A/duck/Vietnam/NCVD-461/2010_H5N1
A/chicken/Vietnam/NCVD-459/2010_H5N1
A/duck/Vietnam/NCVD-891/2010_H5N1
A/duck/Vietnam/NCVD-890/2010_H5N1
0.0010

Fig.S7

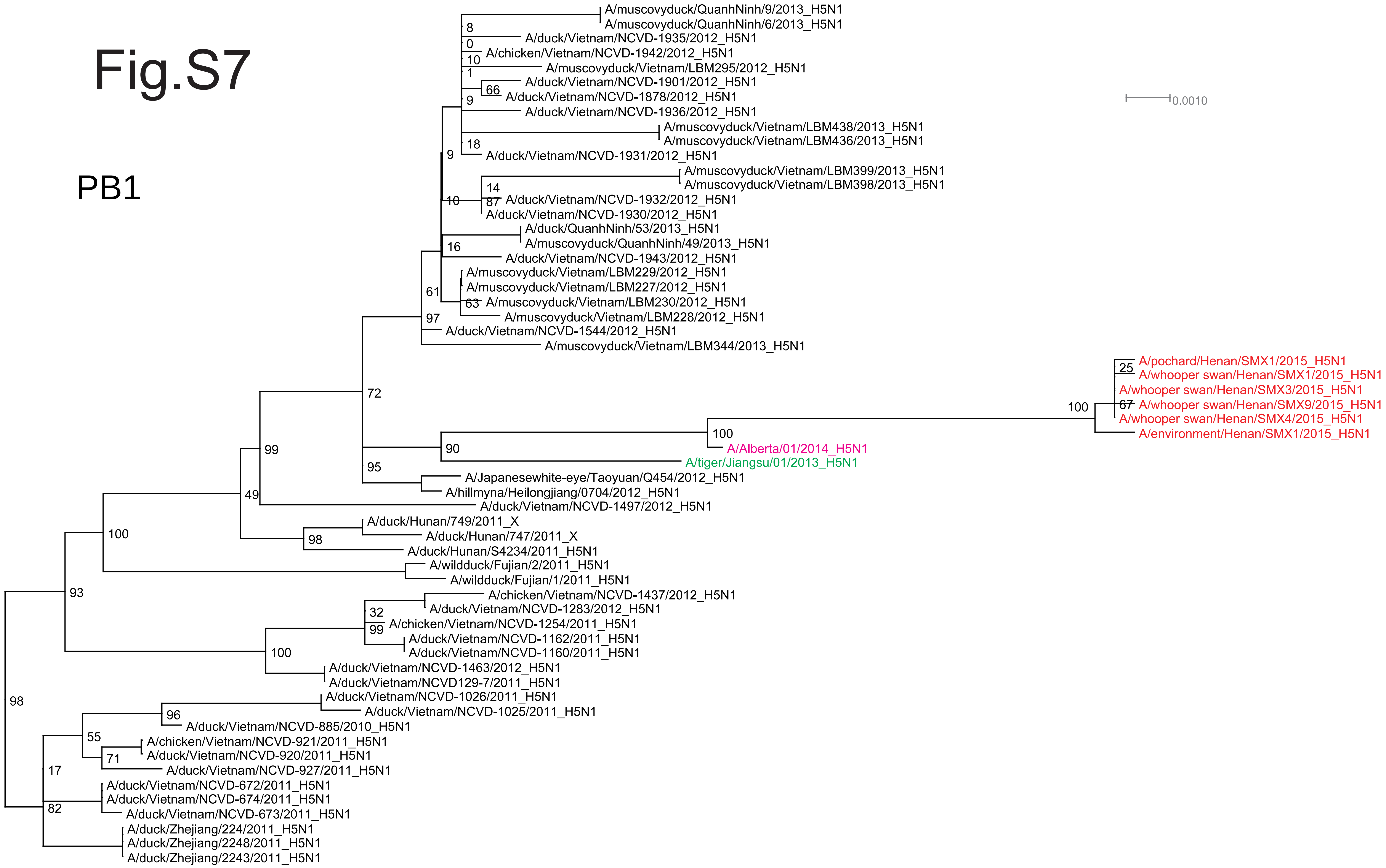
PB1
0.0010
A/muscovyduck/QuanhNinh/9/2013_H5N1
A/muscovyduck/QuanhNinh/6/2013_H5N1
A/duck/Vietnam/NCVD-1935/2012_H5N1
A/chicken/Vietnam/NCVD-1942/2012_H5N1
A/muscovyduck/Vietnam/LBM295/2012_H5N1
A/duck/Vietnam/NCVD-1901/2012_H5N1
A/duck/Vietnam/NCVD-1878/2012_H5N1
A/duck/Vietnam/NCVD-1936/2012_H5N1
A/muscovyduck/Vietnam/LBM438/2013_H5N1
A/muscovyduck/Vietnam/LBM436/2013_H5N1
A/duck/Vietnam/NCVD-1931/2012_H5N1
A/muscovyduck/Vietnam/LBM399/2013_H5N1
A/muscovyduck/Vietnam/LBM398/2013_H5N1
A/duck/Vietnam/NCVD-1932/2012_H5N1
A/duck/Vietnam/NCVD-1930/2012_H5N1
A/duck/QuanhNinh/53/2013_H5N1
A/muscovyduck/QuanhNinh/49/2013_H5N1
A/duck/Vietnam/NCVD-1943/2012_H5N1
A/muscovyduck/Vietnam/LBM229/2012_H5N1
A/muscovyduck/Vietnam/LBM227/2012_H5N1
A/muscovyduck/Vietnam/LBM230/2012_H5N1
A/muscovyduck/Vietnam/LBM228/2012_H5N1
A/duck/Vietnam/NCVD-1544/2012_H5N1
A/muscovyduck/Vietnam/LBM344/2013_H5N1
A/pochard/Henan/SMX1/2015_H5N1
A/whooper swan/Henan/SMX1/2015_H5N1
A/whooper swan/Henan/SMX3/2015_H5N1
A/whooper swan/Henan/SMX9/2015_H5N1
A/whooper swan/Henan/SMX4/2015_H5N1
A/environment/Henan/SMX1/2015_H5N1
A/Alberta/01/2014_H5N1
A/tiger/Jiangsu/01/2013_H5N1
A/Japanesewhite-eye/Taoyuan/Q454/2012_H5N1
A/hillmyna/Heilongjiang/0704/2012_H5N1
A/duck/Vietnam/NCVD-1497/2012_H5N1
A/duck/Hunan/749/2011_X
A/duck/Hunan/747/2011_X
A/duck/Hunan/S4234/2011_H5N1
A/wildduck/Fujian/2/2011_H5N1
A/wildduck/Fujian/1/2011_H5N1
A/chicken/Vietnam/NCVD-1437/2012_H5N1
A/duck/Vietnam/NCVD-1283/2012_H5N1
A/chicken/Vietnam/NCVD-1254/2011_H5N1
A/duck/Vietnam/NCVD-1162/2011_H5N1
A/duck/Vietnam/NCVD-1160/2011_H5N1
A/duck/Vietnam/NCVD-1463/2012_H5N1
A/duck/Vietnam/NCVD129-7/2011_H5N1
A/duck/Vietnam/NCVD-1026/2011_H5N1
A/duck/Vietnam/NCVD-1025/2011_H5N1
A/duck/Vietnam/NCVD-885/2010_H5N1
A/chicken/Vietnam/NCVD-921/2011_H5N1
A/duck/Vietnam/NCVD-920/2011_H5N1
A/duck/Vietnam/NCVD-927/2011_H5N1
A/duck/Vietnam/NCVD-672/2011_H5N1
A/duck/Vietnam/NCVD-674/2011_H5N1
A/duck/Vietnam/NCVD-673/2011_H5N1
A/duck/Zhejiang/224/2011_H5N1
A/duck/Zhejiang/2248/2011_H5N1
A/duck/Zhejiang/2243/2011_H5N1

Fig.S8

PB2

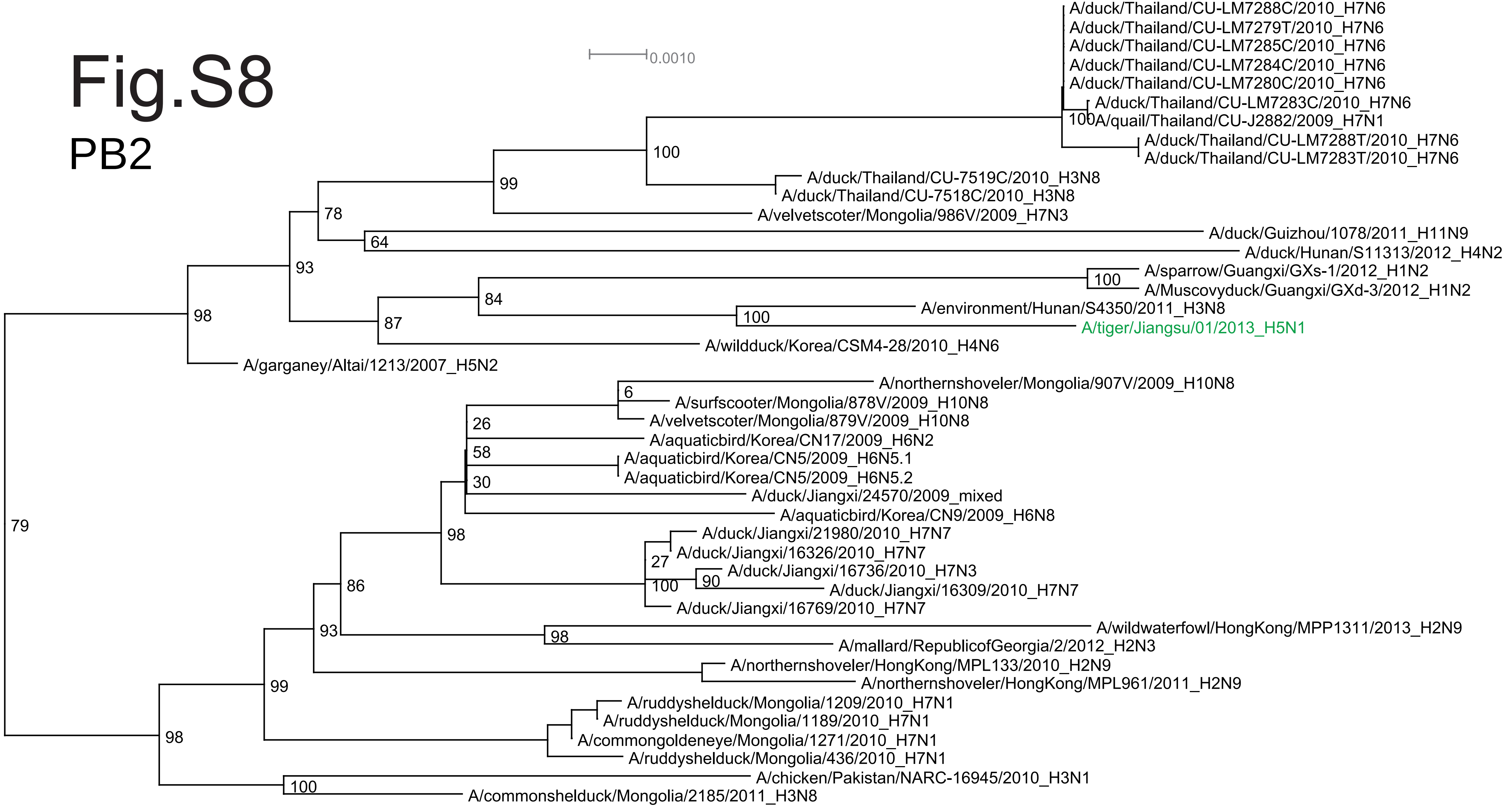

0.0010
A/duck/Thailand/CU-LM7288C/2010_H7N6
A/duck/Thailand/CU-LM7279T/2010_H7N6
A/duck/Thailand/CU-LM7285C/2010_H7N6
A/duck/Thailand/CU-LM7284C/2010_H7N6
A/duck/Thailand/CU-LM7280C/2010_H7N6
A/duck/Thailand/CU-LM7283C/2010_H7N6
100
A/quail/Thailand/CU-J2882/2009_H7N1
A/duck/Thailand/CU-LM7288T/2010_H7N6
A/duck/Thailand/CU-LM7283T/2010_H7N6
100
A/duck/Thailand/CU-7519C/2010_H3N8
A/duck/Thailand/CU-7518C/2010_H3N8
99
A/velvetscoter/Mongolia/986V/2009_H7N3
78
A/duck/Guizhou/1078/2011_H11N9
64
A/duck/Hunan/S11313/2012_H4N2
93
A/sparrow/Guangxi/GXs-1/2012_H1N2
100
A/Muscovyduck/Guangxi/GXd-3/2012_H1N2
84
A/environment/Hunan/S4350/2011_H3N8
100
A/tiger/Jiangsu/01/2013_H5N1
98
87
A/wildduck/Korea/CSM4-28/2010_H4N6
A/garganey/Altai/1213/2007_H5N2
A/northernshoveler/Mongolia/907V/2009_H10N8
6
A/surfscooter/Mongolia/878V/2009_H10N8
A/velvetscoter/Mongolia/879V/2009_H10N8
26
A/aquaticbird/Korea/CN17/2009_H6N2
58
A/aquaticbird/Korea/CN5/2009_H6N5.1
A/aquaticbird/Korea/CN5/2009_H6N5.2
30
A/duck/Jiangxi/24570/2009_mixed
A/aquaticbird/Korea/CN9/2009_H6N8
79
A/duck/Jiangxi/21980/2010_H7N7
98
A/duck/Jiangxi/16326/2010_H7N7
27
A/duck/Jiangxi/16736/2010_H7N3
90
86
100
A/duck/Jiangxi/16309/2010_H7N7
A/duck/Jiangxi/16769/2010_H7N7
A/wildwaterfowl/HongKong/MPP1311/2013_H2N9
98
93
A/mallard/RepublicofGeorgia/2/2012_H2N3
A/northernshoveler/HongKong/MPL133/2010_H2N9
A/northernshoveler/HongKong/MPL961/2011_H2N9
99
A/ruddyshelduck/Mongolia/1209/2010_H7N1
A/ruddyshelduck/Mongolia/1189/2010_H7N1
98
A/commongoldeneye/Mongolia/1271/2010_H7N1
A/ruddyshelduck/Mongolia/436/2010_H7N1
A/chicken/Pakistan/NARC-16945/2010_H3N1
100
A/commonshelduck/Mongolia/2185/2011_H3N8
